# Targeted and personalized immunotherapy in lung adenocarcinoma: single-cell RNA sequencing of *MAFF*+ tumor cells and the therapeutic potential of *FOS*

**DOI:** 10.3389/fimmu.2025.1649147

**Published:** 2025-08-27

**Authors:** Xiangsong Cheng, Shu Chen, Yilong Fu, Runze Jiang, Yanlong Jing, Bizhu Zhao, Dong Guo, Liangyu Wang, Zi Ye, Yumeng Li, Xianliang Chen

**Affiliations:** ^1^ Department of Respiratory Medicine, Heart Center, Henan Provincial People’s Hospital, Central China Fuwai Hospital of Zhengzhou University, Fuwai Central China Cardiovascular Hospital & Central China Branch of National Center for Cardiovascular Diseases, Zhengzhou, Henan, China; ^2^ School of Basic Medical Sciences, Xinxiang Medical University, Xinxiang, Henan, China; ^3^ Clinical Medicine, The First Clinical School of Zhengzhou University, Zhengzhou, Henan, China; ^4^ Shandong University of Traditional Chinese Medicine, Jinan, Shandong, China; ^5^ Department of Scientific Research, The First Affiliated Hospital of Zhengzhou University, Zhengzhou, Henan, China

**Keywords:** lung adenocarcinoma, MAFF, tumor microenvironment, *FOS*, immunotherapy, drug sensitivity

## Abstract

**Background:**

Non-small cell lung cancer (NSCLC) was a major cause of cancer-related mortality globally. Despite advancements in immunotherapy and targeted therapies, clinical outcomes were still limited by tumor heterogeneity and treatment resistance. The transcription factor (TF) FOS, a key component of the AP-1 complex, was linked to tumor progression and therapy resistance in various cancers, but its precise mechanisms remained unclear, and its role in lung adenocarcinoma (LUAD) was unknown. We investigated the tumor microenvironment (TME) of LUAD using single-cell RNA sequencing (scRNA-seq) to identify potential therapeutic vulnerabilities and *FOS*-driven mechanisms.

**Methods:**

We identified fourteen cell types by analyzing scRNA-seq data from LUAD samples (GSE164789) using Seurat (v4.4.0) and Harmony for batch correction. InferCNV was utilized to characterize the tumor cell subtypes after they were clustered using marker genes. CytoTRACE and Monocle were used to create pseudotime trajectories in order to map differentiation states. CellChat revealed intercellular communication networks, while SCENIC identified TF regulatory modules. The CCK-8, Edu, Transwell, and wound healing assays showed that *FOS* knockdown functionally validated A549 and NCI-H1975 cells. Furthermore, a prognostic model was developed.

**Results:**

We discovered that invasive LUAD was dominated by a highly stem-like C0 *MAFF+* tumor cell subtype that produced chemokines and activated lipid metabolism. These cells stimulated immunosuppression and tumor-associated macrophage (TAM) differentiation by interacting with macrophages via MIF-(CD74+CD44) signaling. Experiments using *FOS* knockdown demonstrated its role in maintaining invasion, migration, and proliferation. Using the MTRS model, patients were categorized into high- and low-risk cohorts, high-risk patients exhibited unique drug sensitivities. Immunoprofile analysis revealed higher M1 macrophages in high-risk patients, suggesting that *FOS* inhibition could repolarize TAMs and enhance immunotherapies.

**Conclusion:**

Our studies show that *FOS* is a main regulator of C0 *MAFF*+ TCs in LUAD, polarizing macrophages via MIF and rewiring lipid metabolism to support cancer. The MTRS model offers clinical value for risk assessment even if *FOS* inhibition shows promise as a therapeutic approach to raise immunotherapy efficacy. Targeting the *FOS* could cause TME immunosuppression to be disrupted, thus LUAD presents a fresh precision oncology approach.

## Introduction

Across the world, the incidence of lung cancer gradually climbed and it ultimately became the foremost reason for cancer fatalities. In addition, smoking served as a major factor that heightened the risk of developing lung cancer. Chronic pulmonary diseases, pulmonary infections, occupational and environmental exposures, and lifestyle factors were all considered non-tobacco risk factors (1). Roughly one-third to one-half of lung cancer cases experienced dyspnea, which could have resulted from direct malignancy in the airways or from involvement of lung parenchyma or pleura. Patients were also at risk of developing pulmonary embolism, pneumothorax, pleural effusion, and/or pericardial effusion. Other less frequent symptoms included hoarseness brought on by involvement of the recurrent laryngeal nerve and chest pain from local tumor infiltration (2).

Lung adenocarcinoma (LUAD) was the most prevalent histological subtype of non-small cell lung cancer (NSCLC), which accounted for about 85% of patient diagnoses (3). In LUAD, it was further categorized into adenocarcinoma *in situ* (AIS), minimally invasive adenocarcinoma (MIA) and invasive adenocarcinoma (IAC). For AIS or MIA, it was defined that if complete resection was performed, these patients could have a disease-specific survival rate of close to 100% (4). Furthermore, in IAC, a strong interaction induced by TGF-β signaling between cancer cells and the tumor microenvironment (TME) was identified, which was not observed in AIS and MIA, indicating that IAC represented a more malignant phenotype (5).

Surgical treatment was most suitable for the initial phase of lung cancer and was regarded as the best treatment approach (6). Nevertheless, the majority of lung cancer patients were generally detected only when the disease had already advanced to a later stage, which may be linked to a poor prognosis. Clinicians also faced difficulties due to treatment options and prognostic evaluation limitations (7). The substantial problem of drug resistance, whether in low-toxicity targeted therapies or conventional chemotherapy, was one of the causes of the high death rate linked to NSCLC (8). The *EGFR* was found to be a major gene implicated in lung cancer, since mutations were detected in more than 40% of adenocarcinomas. Three drugs were available for the treatment of *EGFR*-mutant cancers. In 2007, researchers discovered a second driver gene that was present in 5-7% of adenocarcinomas. This gene, known as *ALK*, encoded a poorly understood signaling protein that occasionally underwent gene rearrangements, causing persistent activation of the protein. However, the benefits of these targeted therapies were often temporary, with most tumors developing resistance after approximately one year of remission (9). Furthermore, because early-stage LUAD was usually asymptomatic, the diagnosis was frequently delayed, resulting in late-stage diagnoses for the majority of patients (10). Given that a 65% survival rate over five years, only 30% of patients received a stage I cancer diagnosis, in patients with advanced stages, this rate dropped to 5% or 6% (11). The overall survival rate for LUAD patients remained low despite improvements in surgical resection, chemotherapy, radiotherapy, and molecular targeted therapy (12). Immunotherapy, including LUAD, has emerged as a recognized cancer treatment approach in recent years (13). However, because immunotherapy suppressed immune activity in specific TME and caused resistance and adverse reactions, merely a minor fraction of the diseased gained benefits, with individual differences and tumor heterogeneity limiting its effectiveness (14). Due to the heterogeneity of LUAD, developing effective personalized therapies continued to pose a significant challenge (15).

Since *FOS* was identified among the first viral genes, it encoded a leucine zipper protein that could associate with JUN family members by dimerization. Through this process, the AP-1 TF complex was formed, which in turn had a significant impact on tumor cell growth, differentiation, survival, and how cells responded to DNA damage (16, 17). This AP-1 complex promoted tumor development by directly transcriptionally repressing the p53 served as a tumor suppressor (18). Existing studies indicated that *FOS* was potentially utilized as a core genetic target in the therapeutic approach to LUAD (19). *FOS* was shown to disrupt cell polarity and induce epithelial-mesenchymal transition (EMT) in breast cancer. Consequently, this process enhanced the ability of breast cancer cells to invade and metastasize (20). Within tongue carcinoma, more *FOS* was bound and expressed the more serious the tongue lesions were (21). Given its proven functions in other malignancies, *FOS* may also be essential for the evolution of LUAD.

A potent technique for researching cell biology at a never-before-seen level of resolution is single-cell RNA sequencing (scRNA-seq). It allowed scientists not only to analyze the heterogeneity of cells but also to detect rare, significant cell types while simultaneously exploring the interactions and communication occurring between different cells. Its wide range of uses included both fundamental and applied research fields (22). In order to clarify the cellular heterogeneity and features of the TME, we carried out a scRNA-seq investigation on LUAD cells. New therapeutic ideas developed from this research contributed to improve patient survival rates and prognosis. This investigation aims at investigating the function of FOS in LUAD with an eye toward the requirement of looking at the molecular pathways supporting treatment resistance and tailored therapeutic methods.

## Materials and methods

### Origination of data

To investigate the TME of LUAD, we analyzed scRNA-seq data (23, 24) obtained from the GEO database (https://www.ncbi.nlm.nih.gov/geo/) (GSE164789). In addition to analyzing gene expression patterns, the study combined clinical information and mutation data, which together provided a more comprehensive foundation for the analysis (25). Since the data were publicly accessible, ethical approval was not required.

### Processed and visualized raw data

The Seurat package (v4.4.0), along with R software (v4.3.3), was employed to process the raw gene expression data, thereby facilitating robust analysis (26). The DoubletFinder package (v2.0.3) was first employed to effectively eliminate possible doublet cells while also filtering out cells of low quality. To eliminate low-quality cells, we applied stringent filtering criteria: cells with nFeature (number of detected genes) outside the range of 300-6,000 or nCount (total number of counts) beyond 500-75,000 were excluded. Eliminated also were cells with mitochondrial gene expression more than 25% or red blood cell gene expression more than 5%.

The “Normalize Data” function in Seurat helped us to normalize the data, subsequently, the “Find Variable Features” tool found 2,000 extremely highly variable genes (27). The “ScaleData” tool helped to further standardize gene expression counts so suited for principal component analysis. Harmony R package (v1.2.0) lower sample batch effects (28). Moreover, the “Cell Cycle Scoring” tool indicated cell cycle phases to ensure appropriate characterizing of cellular states (29). For dimensionality reduction, clustering analyses were performed and gene expression patterns were visualized using UMAP (30, 31).

### Classification of cell subtypes

Cell subtypes were determined by performing clustering analysis in Seurat, utilizing the “FindNeighbors” and subsequently the “FindClusters” functions (32, 33). The diverse cell subtypes within the TME were accurately classified because clusters were annotated according to the average expression levels of established marker genes.

### Assessment of cell stemness

Stemness of cells was evaluated by computing gene set activity scores from scRNA-seq data using the AUCell method. Gene sets might be ranked based on degree of enrichment inside individual cells. This approach defined the variations in tumor cell subgroups and their stemness characteristics.

### Trajectory analysis of tumor cell subtypes

We used CytoTRACE, a computational tool for predicting developmental potential, to esitmate the differentiation status of tumor cell subtypes (34). Pseudotime trajectories were created from Monocle (v2.24.1), while lineage architecture was deduced from Slingshot (v2.8.0), which used a cluster-based minimal spanning tree (MST) (35). Smooth trajectory curves produced by the “getLineages” and “getCurves” let differentiation paths and branching events be seen.

### Enrichment studies of cellular subtypes

Using “FindAllMarkers,” differentially expressed genes (DEGs) were found in Seurat. We investigated their biological relevance using Genomic Variant Analysis (GSVA) and Gene Set Enrichment Analysis (GSEA). Kyoto Encyclopedia of Genes and Genomes (KEGG), Gene Ontology (GO), and ClusterProfiler (v4.8.2) derived functional annotations (36, 37). Using an adjusted P-value threshold of 0.05, we detected a range of GO terms that showed significant enrichment, thereby helping to elucidate the regulatory processes associated with various tumor cell subtypes (38, 39).

### Analysis of interactions among cells

The CellChat R package (v1.6.1) was used to analyze cellular communications within the TME (40). The “IdentifyCommunicationPatterns” function measured discrete communication patterns, and the variations in the intensity of interactions between cells were analyzed using the “netVisual diffInteraction” (41). This analysis revealed key signaling pathways and ligand-receptor pairs mediating cellular crosstalk in LUAD. To visually examine the signals that enter and exit every cell, we utilized scatter plots, heatmaps, and a range of other visualization techniques. Moreover, we considered associations among different cell types to be meaningful when the P value was less than 0.05.

### Single-cell regulatory network inference and clustering investigation

We built clusters of TCs and single-cell regulatory networks using Python (v3.9.19) and the pySCENIC library (v0.12.1) (42). Using data on rankings of human gene motif from (https://resources.aertslab.org/cistarget/), we determined the top five TFs displaying the most notable expression changes.

### Established and assessed a prognostic prediction model

The central goal of this investigation was to determine how effectively specific genes associated with various LUAD subtypes could predict patient survival outcomes. After identifying the most significant prognostic genes, we demonstrated that they served as strong predictors for constructing reliable prognostic models (43). This identification process was carried out through both univariate and multivariate Cox proportional hazards analyses (44). Thereafter, we employed a risk evaluation technique, in which the final risk value was obtained by adding up the multiplied values of gene expression amounts and their matching coefficients: $\text{Risk score}=\sum_{\text{i}}^{\text{n}}\text{Xi}\times \text{Yi}$ . Furthermore, we derived optimal cut-off values using the “surv_cutpoint” function, which enabled a comparative analysis of prognostic differences among patient subgroups. Survival analysis was subsequently conducted by means of the “Survival” package in R (v4.3.3), and survival curves were also constructed using the “ggsurvplot” function so as to examine the predictive accuracy of our risk model (45). In addition, we assessed the model’s reliability by constructing ROC curves (46) with the package of “timeROC” (v0.4.0), thus offering a thorough assessment of both the model’s accuracy and its calibration.

### Analysis of immune microenvironment

To comprehensively analyze the immune landscape, we first used the CIBERSORT R software package (v0.1.0) to estimate immune cell scores for each patient (47, 48). Subsequently, we investigated the infiltration of immune cells in detail and also evaluated the differential expression of immune checkpoint-related genes (49). We further examined associations among risk scores, immune cell populations, and genes included in the model. Additionally, we made use of the Tumor Immune Dysfunction and Rejection (TIDE) platform (http://tide.dfci.harvard.edu) to predict patient responses to tumor immunotherapy.

### Identification of malignant cells by inferCNV

The inferCNV R software package (v1.16.0, https://github.com/broadinstitute/inferCNV) served as our primary tool for CNV inference, enabling us to characterize the CNV patterns within multiple cell subtypes using the inferCNV algorithm. We assessed both relative gene expression and chromosome location data to infer CNV status across individual cells (50).

### Analysis of drug sensitivity

To strengthen the clinical significance of our results regarding drug applications, we evaluated the sensitivity of multiple agents. Specifically, we employed the “pRRophetic” package (v0.5) to estimate the half-maximal inhibitory concentration (IC50) for every individual case and subsequently compared drug sensitivities across the high-risk and low-risk groups.

### Cellular culture

The A549 cell strain was cultured in F-12K medium under standardized conditions, which included a temperature of 37 °C, 5% CO_2_, and 95% humidity. The medium was fortified by incorporating 10% fetal calf serum along with 1% antimicrobials. Likewise, the NCI-H1975 cell strains were routinely preserved in RPMI-1640 medium with similar environmental conditions, and this medium also contained 10% fetal calf serum as well as 1% antimicrobials to ensure the cells attained their best growth rates.

### Transfection of the cells

A decrease in *FOS* expression was observed, which was partly a result of RNA synthesized by GenePharma (Suzhou, China). After introducing the *FOS*-targeting siRNAs (si*FOS*-1 and si*FOS*-2) combined with a control siRNA (si-NC), cells were distributed into 6- well chambers at a coverage level of 50%. Transfection was carried out in accordance with the instructions from the manufacturer, utilizing Lipofectamine 3000RNAiMAX (Invitrogen, USA). Each siRNA (RIbbio, China) was individually introduced into the cells.

### Western blotting

Following achievement of 70% confluence in transfected cells, lysis was conducted with RIPA buffer. The lysates underwent centrifugation at 12,000 rpm for 15 minutes to clarify the extracts before being processed by SDS-PAGE. The separated proteins were then transferred onto a PVDF membrane, which was blocked at room temperature for 1.5 hours with 5% calf serum albumin. The membrane underwent incubation throughout the night at 4°C with an anti-FOS antibody, followed by a one-hour incubation with a secondary antibody. Finally, the presence of FOS protein was revealed using a chemiluminescent Western blot substrate.

### Quantitative real-time polymerase chain reaction

To extract RNA, cell lysis was achieved with Trizol reagent. All water sources, laboratory instruments, and workspaces were maintained RNase-free throughout the protocol to ensure RNA stability. Once RNA was isolated, the PrimeScript™ reagent kit was used for reverse transcription. Finally, quantitative real-time PCR was conducted, using SYBR Green master mix as the fluorescent indicator for the detection of amplification.

### Assay of the viability of the cells

To evaluate the survival of A549 and NCI-H1975 cells following transfection, the Cell Counting Kit-8 (CCK-8) method was applied (51). The cells were plated into 96-well chambers at 5 × 10³ cells per well and, following this, were left to adhere for 24 hours. Afterwards, every well gained 10 µL of CCK-8 reagent (A311-01, Vazyme), followed by an additional 2-hour incubation at 37°C in the absence of light. Light absorption at 450 nm was recorded each day from the first to the fourth day by means of a microtiter reader (A33978, Thermo). The collected light density data were averaged and graphed to illustrate changes in cell viability over the time course.

### Experiments of 5-Ethynyl-2’-deoxyuridine proliferation

After the transfection procedure, A549 and NCI-H1975 cells were plated into 6-well chambers at 5 × 10³ cells per well. Cells were allowed to incubate for 24 hours at ambient temperature before adding the EdU working solution for another 2-hour period. Being rinsed two times with PBS, the cells were fixed in 4% paraformaldehyde for a duration of 15 minutes. After fixation, permeabilization was performed, and the cells were quenched for 15 minutes in a solution containing 0.5% Triton X-100 and 2 mg/ml glycine. Following this, 1 ml of Apollo solution and 1 ml of Hoechst stain were applied, and the cells were incubated for 30 minutes. Finally, fluorescence microscopy was employed at the end to image the cells and evaluate their proliferation.

### Transwell assay

Initially, cells were deprived of serum for 24 hours by incubating them in a serum-free medium. Following this, the cellular suspension was blended with Matrigel (BD Biosciences, USA) and the upper well of the Costar plate was loaded with the cell suspension, while the lower well received serum-containing medium to form a chemotactic gradient. Then cells were allowed to migrate and invade in a constant-temperature apparatus for a duration of 48 hours. Afterwards, 4% paraformaldehyde solution served to fix the cells, and crystal violet staining was employed to evaluate their invasion capacity visually.

### Wound healing assay

Cells with stable transfection were cultured in 6- well chambers and incubated at 37°C within a moist environment supplemented with 5% CO_2_ until they became confluent. Sterile 200 µL pipette tips were then used to create straight scratches in the cell monolayer. The wells were carefully washed with PBS to remove floating cells and debris. Following this, the cells were incubated with serum-free medium to facilitate migration. Photographs were captured at both the beginning (0 hours) and after 48 hours, and scratch widths were measured using Image-J for subsequent analysis.

### Statistical procedures

The datasets were subjected to analysis using R (v4.3.3) and Python (v3.9.19). For comparisons between groups, Wilcoxon’s test and Pearson’s correlation coefficient were applied (52). We interpreted the significance at the statistical level following the criteria listed below: *P< 0.05, **P< 0.01, ***P< 0.001, and ****P< 0.0001, “ns” was used to denote results without statistical significance. These analytical methods and cutoffs were adopted to confirm the robustness of the findings.

## Results

### Single cell landscape of LUAD

**Figure 1:**
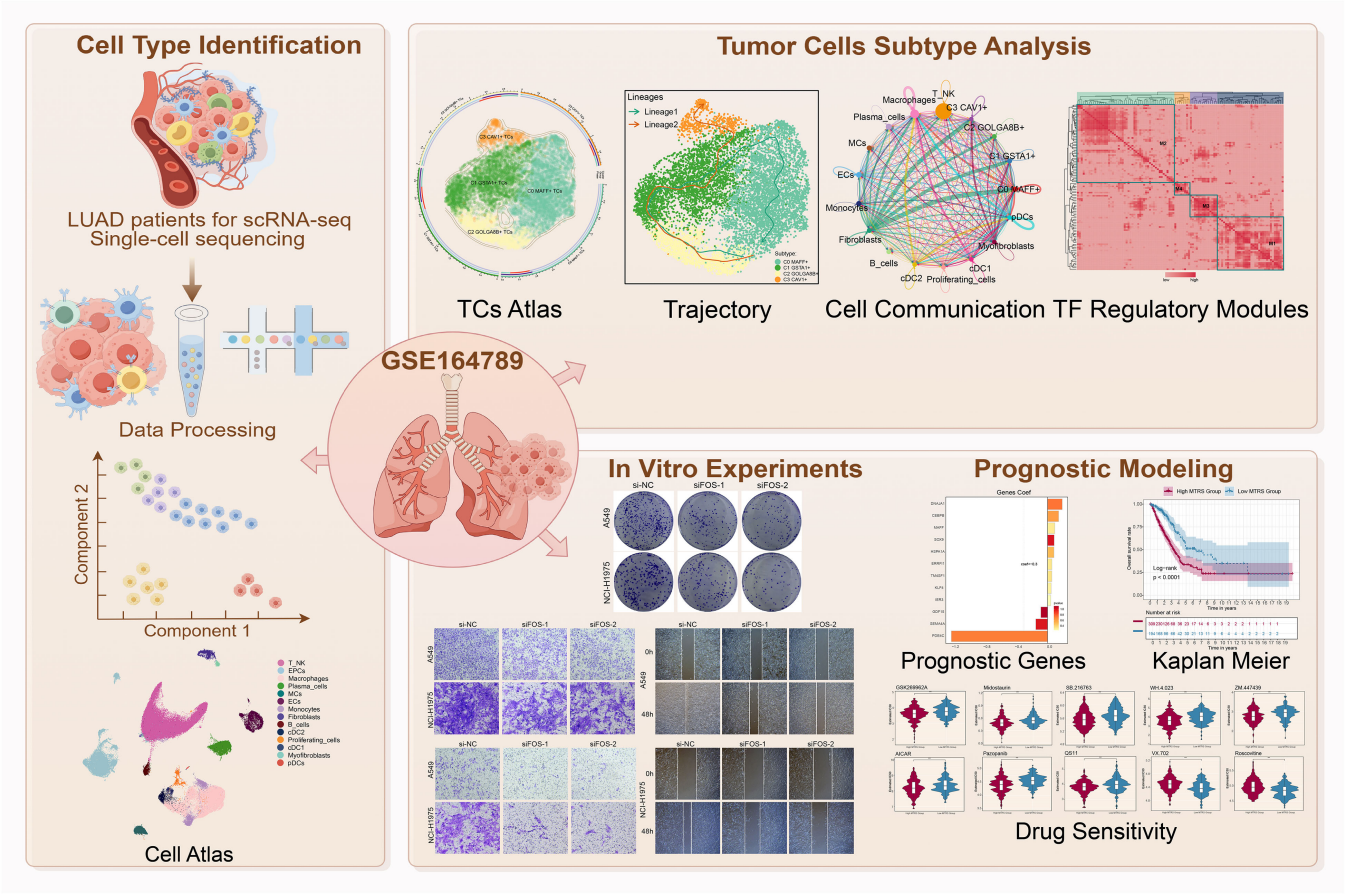
Graphical abstract. Workflow demonstrated LUAD single-cell RNA sequencing analysis of the GSE 164789 dataset.

**Figure 2:**
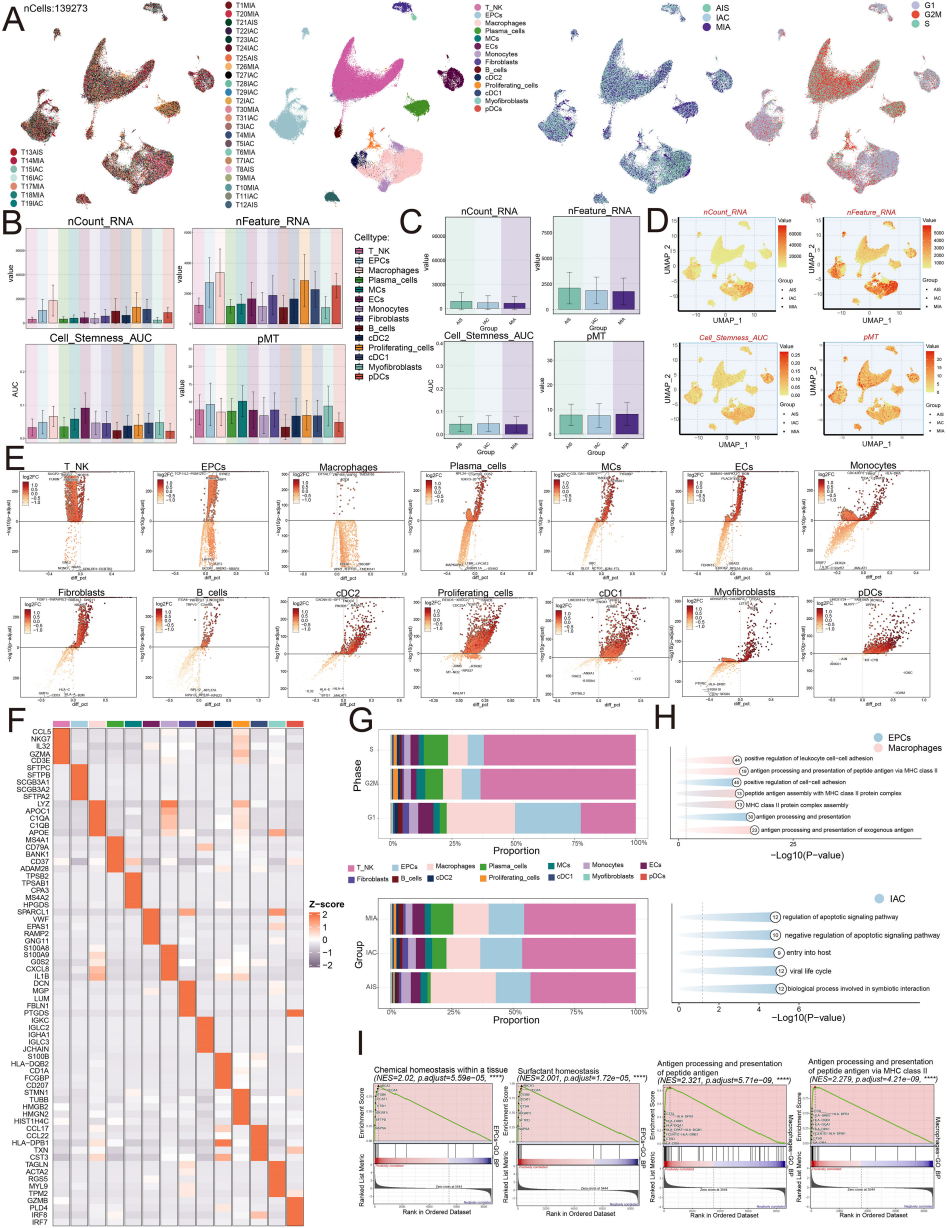
Single-cell profiling of LUAD. **(A)** The UMAP plots mapped out the distribution of thirty-one samples, and additionally portrayed the allocation of fourteen unique cell types—including T and NK cells, EPCs, Macrophages, Plasma cells, MCs, ECs, Monocytes, Fibroblasts, B cells, cDC2, Proliferating cells, cDC1, Myofibroblasts, and pDCs—across the total cell population. In addition, these plots provided a visual of the group distributions and various cell cycle phases (from left to right). **(B)** The bar graphs represented the levels of nCount RNA, nFeature RNA, cell stemness AUC, and pMT for each of the fourteen cell types. **(C)** Bar graphs were utilized to display the expression analyses of three groups in terms of nCount RNA, nFeature RNA, cell stemness AUC, and pMT. **(D)** The UMAP plots depicted the distribution of nCount RNA, nFeature RNA, cell stemness AUC, and pMT, with each group identified by different symbols. **(E)** Volcano plots were used to present the five most significantly upregulated and downregulated genes across the fourteen distinct cell types. **(F)** The heatmap presented the distribution of the top five marker gene expressions among diverse cell populations. **(G)** The distribution of cell types across various phases and groups was visualized using stacked bar graphs**. (H)** Enrichment analysis was visualized for EPCs, Macrophages, and the IAC group. **(I)** GSEA enrichment analysis revealed the upregulated pathways in EPCs and Macrophages.

To explore the ECs, we analyzed their CNVs using inferCNV with ECs as a reference (**Supplementary Figure 1**). We examined the obtained dataset to explore the single-cell profile present in the LUAD environment. **Figure 1** illustrated our procedure. By examining five localized adenocarcinomas and twenty-six infiltrating adenocarcinomas from GSE164789, we employed dimensionality reduction clustering with UMAP plots. This analysis initially exhibited how thirty-one individual samples were distributed and subsequently identified fourteen cell types: T and NK cells, epithelial cells (EPCs), macrophages, plasma cells, mesenchymal cells (MCs), endothelial cells (ECs), monocytes, fibroblasts, B cells, conventional dendritic cells type 2 (cDC2), proliferating cells, conventional dendritic cells type 1 (cDC1), myofibroblasts, and plasmacytoid dendritic cells (pDCs). Furthermore, we presented the distribution of cells within three groups (AIS, IAC, MIA) and across three different phases of the cell cycle (G1, G2/M, S) (**Figure 2A**). We examined the expression profiles of nCount RNA, nFeature RNA, cell stemness AUC, and pMT among the fourteen cell types and three different groups (**Figures 2B, C**). Additionally, **Figure 2D** illustrated the cellular distribution of the dataset. Volcano plots revealed the presence of different genes among these cell types (**Figure 2E**). In EPCs, *TCP11L2, POM121C, HYAL2, HBP1*, and *SYNE2* were found to be upregulated, while *BCOR, LHFPL6, IKZF2, SRPK1*, and *SRSF8* exhibited downregulation. In macrophages, *EIF5AL1, ZNF468, AGO1, USP25*, and *TMEM165* showed upregulation, whereas *UPRT, PDHX, MTFR1L, TRIOBP*, and *TMEM141* showed downregulation. We also provided the top five marker genes corresponding to the fourteen cell types, as depicted in **Figure 2F**. Through investigating the ratios of individual cell types in the three groups alongside cell cycle data, we found that each cell type distributed across separate phases within the cell cycle, with EPCs and macrophages constituting the main components of the IAC group, predominantly located in the differentiated G1 phase (**Figure 2G**).

We then conducted functional enrichment analysis. The analysis suggested that IAC group displayed increased activity in pathways connected to the regulation of apoptotic signaling, negative regulation of apoptotic signaling, entry into the host, viral life cycle, and biological processes involved in symbiotic interaction. EPCs indicated enrichment within pathways related to positive regulation of cell-cell adhesion and antigen processing and presentation, while macrophages were enriched in pathways associated with positive regulation of leukocyte cell-cell adhesion, antigen processing and presentation of peptide antigens via MHC class II, peptide antigen assembly with MHC class II protein complex, assembly of MHC class II protein complexes, and antigen processing and presentation of exogenous antigens (**Figure 2H**). Furthermore, EPCs demonstrated upregulation in pathways related to chemical homeostasis within tissues and surfactant homeostasis, whereas macrophages displayed significant upregulation in antigen processing and presentation of peptide antigens and antigen processing and presentation of peptide antigens via MHC class II (**Figure 2I**).

### Visualization analysis of LUAD tumor cell subtypes

**Figure 3:**
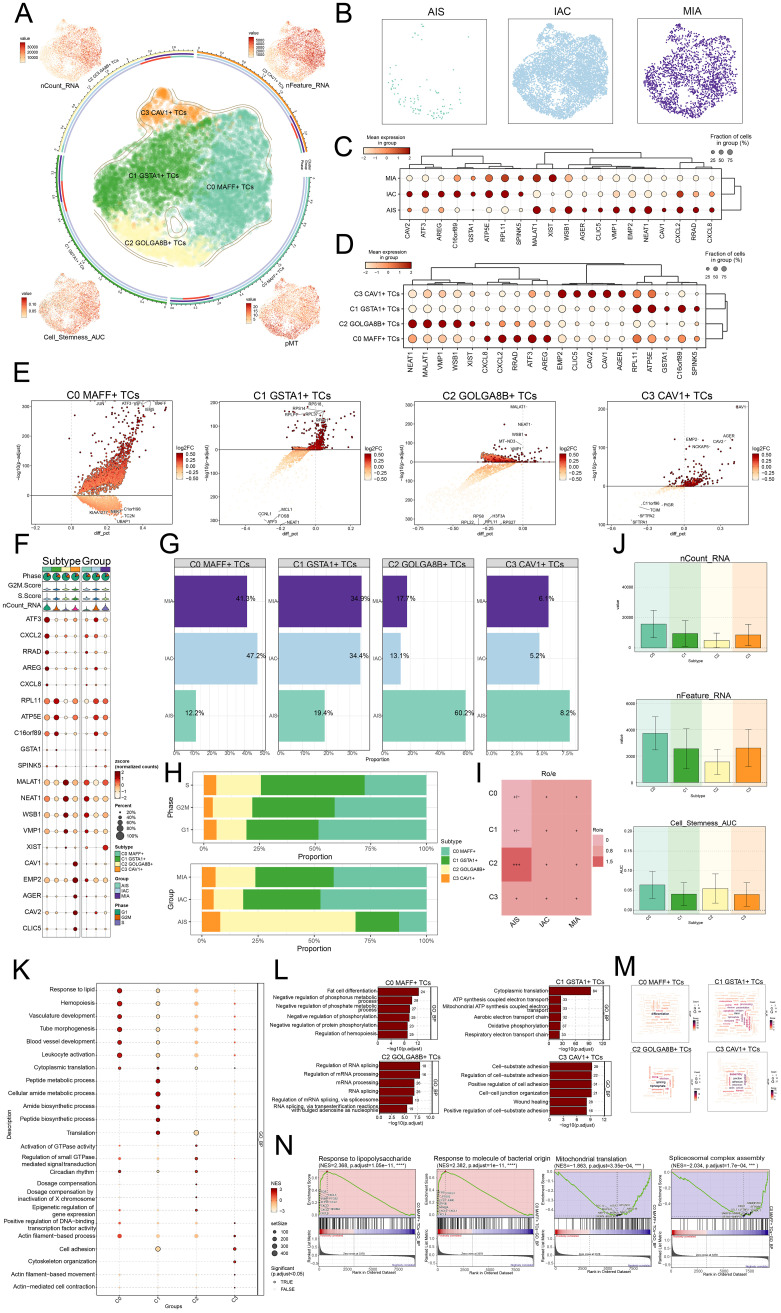
*MAFF+* TCs specifically expressed in malignant EPCs and enrichment analysis in TCs of LUAD. **(A)** The arrangement of four distinct tumor cell subtypes in LUAD was shown using a circular plot, with boundary curves outlining each subtype. The total cell numbers within each class were represented on the outer axis using a logarithmic scale. UMAP plots placed at each of the four corners, moving clockwise from the top left, visualized the distribution of nCount RNA, nFeature RNA, pMT, and cell stemness AUC among all TCs **(B)** The UMAP plots also revealed how TCs were distributed among AIS, IAC, and MIA. **(C)** The bubble plot displayed the marker gene expression patterns within three groups. **(D)** The bubble plot presented the marker gene profiles in the four tumor cell subtypes, with bubble diameter corresponding to the percentage of cells with expression and color reflecting the normalized measurement. **(E)** Volcano plots displayed the most significantly upregulated and downregulated genes in each of the four subtypes. **(F)** For each subtype, the mean values of the top five differentially expressed genes were determined were also depicted using bubble plots, where bubble size and color denoted expression percentage and data normalization, respectively. **(G)** Bar graphs visualized the percentages of different groups within each tumor cell subtype. **(H)** Stacked bar graphs were also used to visualize the allocation of these subtypes within different phases and groups. **(I)** The group preference for each subtype was evaluated with the Ro/e score. **(J)** Bar graphs presented the expression levels of nCount RNA, nFeature RNA, and cell stemness AUC across the subtypes. **(K)** The GSEA results for each subtype were visualized in a bubble plot. **(L)** Bar graphs provided insights into biological processes across the subtypes. **(M)** Biological processes associated with each subtype were shown via word cloud graphs. **(N)** GSEA was used to analyze both positively and negatively enriched pathways in C0 *MAFF*+ TCs.

The involvement of the TME in tumorigenesis became widely recognized, as it harbored TCs that, through their interactions with other cells via the circulatory and lymphatic networks, played an essential part in both the emergence and advancement of cancer (53). We characterized the significance of TCs in the TME. Four subtypes of LUAD TCs were classified according to the expression of marker genes: C0 *MAFF+* TCs, C1 *GSTA1+* TCs, C2 *GOLGA8B+* TC, and C3 *CAV1+* TCs, and demonstrated the nCount RNA, nFeature RNA, cell stemness AUC, and pMT for each subtype (**Figure 3A**). Subsequently, we examined the primary distribution of TCs across different groups using UMAP plots (**Figure 3B**). Bubble plots illustrated the marker genes associated with various groups and tumor cell subtypes (**Figures 3C, D**). In MIA, we found that *ATP5E, RPL11, SPINK5, MALAT1*, and *XIST* were highly expressed. In AIS, *MALAT1, WSB1, VMP1, EMP2*, and *NEAT1* exhibited high expression levels. In IAC, *ATF3, AREG, C16orf89, ATP5E*, and *RPL11* showed elevated expression. The C0 subtype exhibited high expression of *CXCL8, CXCL2, RRAD, ATF3*, and *AREG.* The C1 subtype showed elevated levels of *RPL11, ATP5E, GSTA1, C16orf89*, and *SPINK5*. The C2 subtype had increased expression of *NEAT1, MALAT1, VMP1, WSB1*, and *XIST*. The C3 subtype displayed high expression of *EMP2, CLIC5, CAV2, CAV1*, and *AGER*. **Figure 3E** visualized these different genes. We found that *JUN, ATF3, IRF1, IER5*, and *MAFF* were upregulated in the C0 subtype. *RPS1, RPL11, RPS14, RPS18*, and *RPL37* were upregulated in the C1 subtype. *MALAT1, NEAT1, WSB1, MT-ND3*, and *VMP1* showed increased expression in the C2 subtype. *CAV1, CAV2, AGER, EMP2*, and *NCKAP5* were upregulated in the C3 subtype. We subsequently analyzed how the top five marker genes were differentially expressed (**Figure 3F**).

Moreover, ratios within separate groups, phases of development, and specific cell subtypes were determined, discovering that the C0 subtype represented the highest proportion within the IAC, accounting for up to 47.2% (**Figures 3G-I**). Therefore, the heterogeneity of the IAC group might be related to the C0 subtype.

Next, we presented the results of nCount RNA, nFeature RNA, and cell stemness AUC for different subtypes using bar graphs (**Figure 3J**). The findings suggested that, in these aspects, C0 demonstrated elevated levels of expression in these metrics relative to the other subtypes. Consequently, we inferred that the C0 subtype likely corresponded to a higher degree of malignancy.

### Analysis of enrichment in LUAD TCs

To explore the biological functions of TCs in LUAD, we conducted GSEA analysis across different subtypes. We observed that the C0 subtype was primarily enriched in the response to lipid (**Figure 3K**). Subsequently, we analyzed the biological processes associated with the four subtypes and the C0 subtype was primarily identified as being enriched in fat cell differentiation, the C1 subtype in cytoplasmic translation, the C2 subtype in regulation of RNA splicing, and the C3 subtype in cell-substrate adhesion (**Figure 3L**). With regard to the biological processes, we found that the C0 subtype was associated with differentiation (**Figure 3M**). While showing considerable negative regulation in pathways linked to mitochondrial translation and spliceosomal complex assembly, the GSEA results also revealed that the C0 subtype displayed a significant positive regulation in pathways related with response to lipopolysaccharide and response to molecules of bacterial origin (**Figure 3N**). C0 subtype linked responses to lipid and molecules of bacterial origin, fat cell differentiation, and hemopoiesis. These results greatly affected the viability and growth of TCs, so facilitating the advancement of LUAD.

### Pseudotime analysis revealed the heterogeneity of stemness and developmental stages among LUAD TCs subtypes

**Figure 4:**
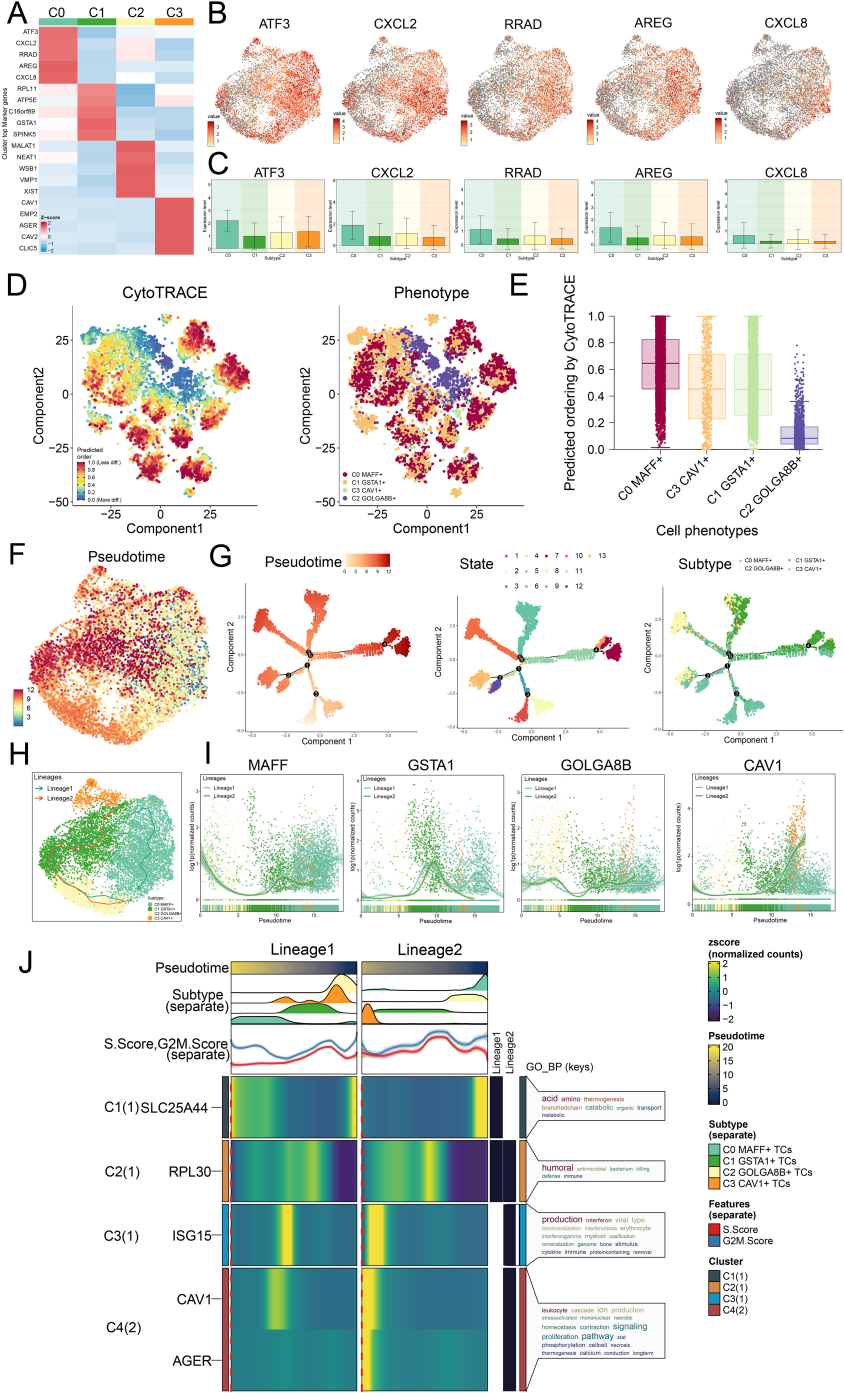
Trajectory analysis on the different tumor cell subtypes. **(A)** Z-scores for stemness-related marker genes across the four subtypes were displayed in a heatmap. **(B)** UMAP plots further mapped the distribution of five cell stemness genes among all TCs. **(C)** Bar graphs illustrated the expression profiles of these five pivotal genes across the four subtypes. **(D)** Left panel depicted the predicted order distribution within TCs as determined by CytoTRACE, with color gradients reflecting the degree of cell stemness. Right panel illustrated the allocation of tumor cell subtypes, each assigned a specific color. **(E)** To rank the stemness among tumor cell subtypes, CytoTRACE analysis was implemented. **(F)** A trajectory analysis was then conducted to map the developmental progression of tumor cell subtypes. **(G)** The differentiation trajectory was color-coded according to pseudotime (left), state (middle), and subtype (right), thereby offering comprehensive insight. **(H)** UMAP plot revealed the trajectory of the differentiation among the four identified subtypes, with C0 *MAFF*+ TCs positioned at the endpoint of lineage 1, solid lines traced the trajectories, and arrows denoted the progression from naive to mature. **(I)** Dynamic trend plots revealed the fluctuations in expression for the four marker genes analyzed. **(J)** Heatmaps displayed the GO enrichment pathways active during tumor cell differentiation, and the top bar graphs annotated both pseudotime and tumor cell subtype. Ridgeline plots outlined the density of subtype distribution across pseudotime, while trajectory plots presented the variation of S.Score (red) and G2/M. Score (blue) as pseudotime progressed.

In our investigation, we examined the expression of genes connected to stemness in the different tumor cell subtypes and determined that the expression levels of *ATF3, CXCL2, RRAD, AREG*, and *CXCL8* were significantly higher in the C0 subtype (**Figure 4A**). UMAP visualization revealed how these genes were distributed (**Figure 4B**). The bar graphs indicated that the expression profiles of *ATF3, CXCL2, RRAD, AREG*, and *CXCL8* in the C0 subtype were expressed at greater levels than in the remaining tumor cell subtypes (**Figure 4C**).

In seeking to understand how LUAD tumor cell subtypes arose and developed, we investigated both their lineage relationships and differentiation states. In **Figures 4D-G**, we analyzed the results of trajectory and CytoTRACE. CytoTRACE analysis indicated that C0 *MAFF+* TCs exhibited a higher degree of stemness (**Figure 4E**). Stemness was closely related to cellular differentiation, higher levels of stemness correspond to lower degrees of differentiation, and TCs in the latter stages of differentiation often possessed greater stemness.

In order to define how the tumor cell subtypes differentiated, we utilized Slingshot analysis studied their developmental trajectories, and further presented the results through UMAP visualization. We first presented two main differentiation trajectories for the four tumor cell subtypes: lineage 1: C2→C1→C0 and lineage 2: C2→C1→C3. The differences between the two trajectories were primarily observed in later stages, where C2 *GOLGA8B*+ TCs were located at the initial stages of differentiation in both lineage 1 and lineage 2, and C0 *MAFF+* TCs were found at the final stage of lineage 1 (**Figure 4H**). Additionally, we analyzed the dynamic trends of marker gene expression among the four subtypes, the high expression of *MAFF+* was predominantly observed at later stages (**Figure 4I**), further validating our Monocle analysis findings.

We ultimately conducted analysis of GO-BP enrichment on the subtypes to verify the biological processes pertinent to both lineages (**Figure 4J**). The dynamic timing approach demonstrated how gene expression in TCs shifted along the two pseudotime trajectories.

### The analysis of intercellular communication along with visualization of the MIF signaling pathway

**Figure 5:**
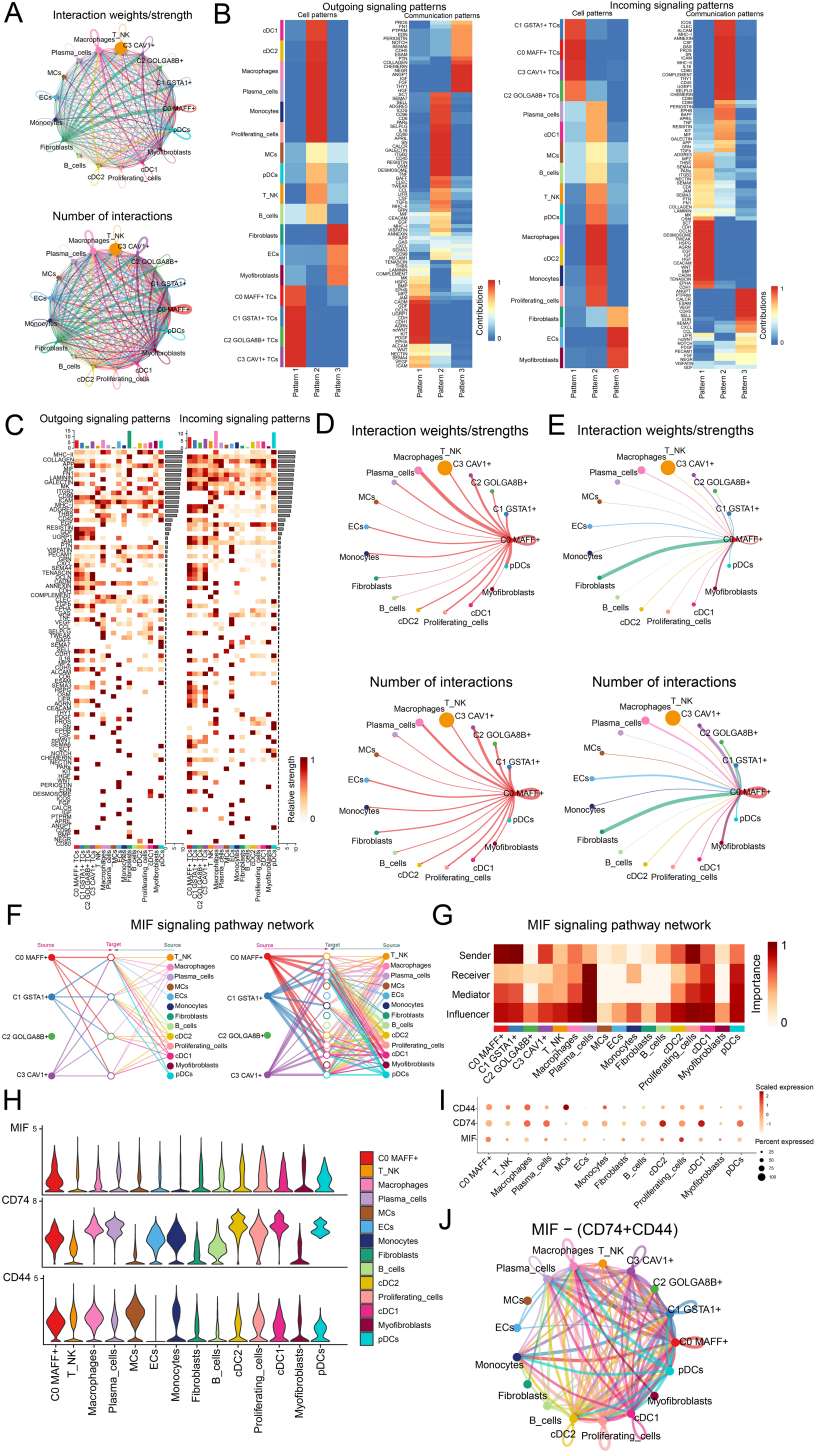
Analysis of the regulatory networks governing genes in C0 *MAFF*+ TCs. **(A)** The circle diagrams provided a summary of both the number and intensity of interactions observed between the four tumor cell subtypes and thirteen different cell types, thus shedding light on their interconnections. **(B)** Separate heatmaps showed the outgoing (left) and incoming (right) signaling effects of the four tumor cell subtypes and the thirteen cell types, as well as the specific input from various proteins within three distinct cell communication patterns. **(C)** Bar graphs were employed to evaluate and compare the outgoing and incoming signaling strengths among four tumor cell subtypes and thirteen types of cells. Meanwhile, the heatmaps provided a visualization of the intensity with which these proteins, implicated in cell communication, were received among the different groups. **(D, E)** These circle diagrams further illustrated the strength (top) and number (bottom) of interactions where C0 *MAFF*+ TCs acted as either the source or the target in relation to other cell types. **(F)** The hierarchical graph depicted the network of interactions between C0 *MAFF*+ TCs and other cell types within the MIF signaling pathway. **(G)** A further heatmap presented the centrality scores for the MIF signaling pathway. **(H, I)** Both the violin plot and bubble plot demonstrated that C0 *MAFF*+ TCs and macrophages potentially interacted through the ligand MIF and the receptors CD74 and CD44. **(J)** Finally, the circle diagram outlined the communication network of MIF-(CD74+CD44) ligand-receptor interactions with TCs receiving the signals.

In order to better comprehend the intricacies of cellular responses, we sought to investigate the networks and intercellular connections that underpin ligand-receptor signaling, which allowed us to depict the interactions among different cell types. By employing analysis of CellChat, we established a comprehensive network of cell-cell interactions covering the majority of cell types, such as C0 *MAFF+* TCs, C1 *GSTA1+* TCs, C2 *GOLGA8B+* TCs, C3 *CAV1+* TCs, T and NK cells, macrophages, plasma cells, MCs, ECs, monocytes, fibroblasts, B cells, cDC2, proliferating cells, cDC1, myofibroblasts and pDCs (**Figure 5A**). We found that in the outgoing signaling pattern, C0 *MAFF*+ TCs predominantly exhibited pattern 1, while JAM, CADM, GDF, OCLN and UGRP1 displayed high expression levels within this pattern. Correspondingly, in the incoming signaling pattern, C0 *MAFF*+ TCs also primarily represented pattern 1, where SCT, CDH, OCLN, DESMOSOME, and TWEAK showed elevated expression levels (**Figure 5B**). The heatmaps illustrated the communication patterns and signaling pathways of each tumor cell subtype (**Figure 5C**). We found that the degree as well as the count of connections involving C0 *MAFF*+ TCs and macrophages were more significant in both afferent and efferent signals, so it could be inferred that there was a strong interaction between C0 *MAFF*+ TCs and macrophages (**Figures 5D, E**).

To present the results more intuitively, we employed a hierarchical graph to depict the relationship between C0 *MAFF+* TCs and macrophages. The results demonstrated that the primary interactions between C0 *MAFF+* TCs and macrophages occurred through paracrine and autocrine signaling, leading to intense communication (**Figure 5F**). Subsequently, through network centrality analysis of the MIF signaling pathway, we examined the roles of C0 *MAFF+* TCs and macrophages within this pathway. The findings revealed that C0 *MAFF+* TCs acted as senders, mediators and influencers, whereas macrophages primarily functioned as influencers, senders and mediators, which could have been linked to the conversion of regular macrophages into tumor-associated macrophages (TAMs) (**Figure 5G**). We compared the receptor-ligand interactions between C0 *MAFF+* TCs and other cell types, allowing us to infer that the MIF ligand from C0 *MAFF+* TCs interacted with the CD74-CD44 receptors on macrophages (**Figures 5H, I**). Further supporting our conclusions was a circular diagram showing the interaction between C0 *MAFF*+ TCs and macrophages within the MIF-(CD74+CD44) signaling pathway (**Figure 5J**).

Overall, our work exposed the interactions between C0 *MAFF*+ TCs and macrophages in LUAD, stressing their possible relationship with the change of macrophages to TAMs, so fostering the development of LUAD.

### TFs directed the tumorigenic mechanisms in C0 *MAFF*+ TCs

TFs governed gene expression through their ability to bind specific consensus sequences located in the local chromatin setting (54). TFs were typically considered to comprise two main domains: a DNA-binding domain and a functional domain. Additionally, these domains were seen as independent and separable, with the DNA-binding domain responsible for gene targeting and the functional domain for facilitating transcriptional regulation (55).

**Figure 6:**
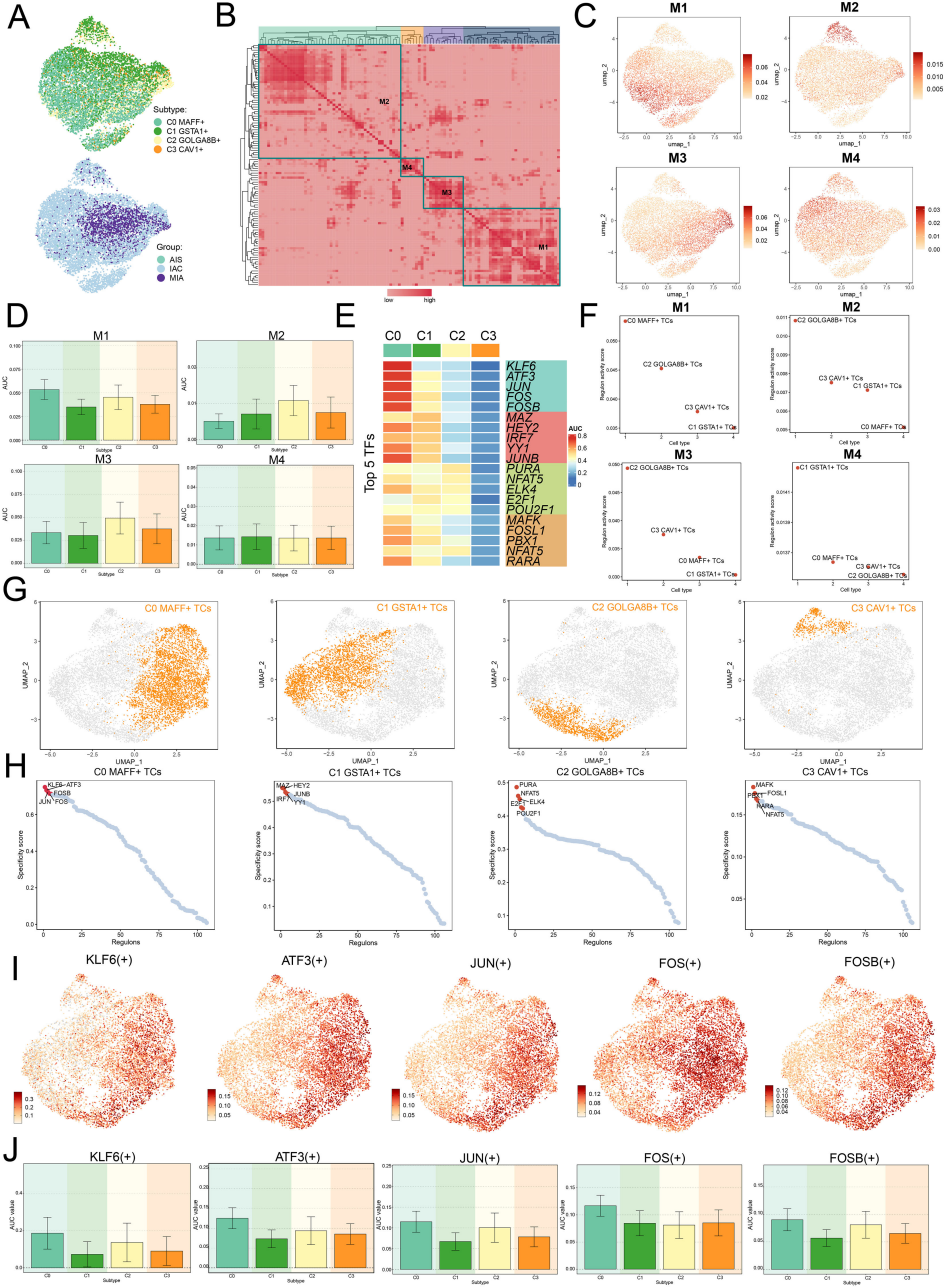
Clustering analysis of TFs and identification of the top five TFs in C0 *MAFF+* TCs. **(A)** UMAP plots visualized all TCs, applying colors determined by regulatory module activity scores and categorizing them by both cell subtypes and group classification. **(B)** Identification of four regulatory modules among various tumor cell subtypes was visualized in the heatmap, relying on SCENIC modules and AUCell similarity assessment. **(C)** UMAP plots further illustrated the unique expression signatures of TFs within the four modules of TCs. **(D)** Bar graphs depicted the AUC values for the four tumor cell subtypes across modules M1, M2, M3, and M4. **(E)** The heatmap highlighted the top five TFs in each of the four tumor cell subtypes. **(F)** Scatter plots ranked the regulatory activity scores of TFs for various tumor cell subtypes in all four modules. **(G, H)** UMAP plots visualized the spatial distribution of each tumor cell, while scatter plots provided the ranking of TFs specificity scores for the top five TFs in every subtype of tumor cell. **(I, J)** UMAP plots exhibited the distribution of selected TFs, and the bar graphs reported the AUC values of the top five TFs in C0 *MAFF*+ TCs across different tumor cell subtypes.

We employed the SCENIC method to perform two-dimensional clustering of TCs from LUAD with reference to various subtypes and groups (**Figure 6A**). Afterwards, we generated a matrix for the connection specificity index based on the similarity of AUCell scores, which categorized LUAD TCs into four regulatory modules: M1, M2, M3, and M4 (**Figure 6B**). A visualization analysis of these modules was conducted, revealing that C0 *MAFF+* TCs exhibited increased expression in the M1 module compared to other subtypes. Furthermore, UMAP visualizations were employed to examine how each tumor cell subtype was distributed among the various modules, indicating that C0 *MAFF+* TCs were predominantly located in the M1 module (**Figures 6C, D**).

Afterwards, we evaluated the top five TFs for each distinct subtype of TCs, with a particular focus on their specificity scores across the various modules. Notably, in M1, C0 *MAFF*+ TCs displayed the highest regulatory activity score, supporting our earlier conclusions (**Figures 6E, F**). Furthermore, we ranked the TFs for each subtype and looked at the distribution of various subtypes (**Figures 6G, H**). In C0 *MAFF*+ TCs, we examined the distribution and expression levels of five important TFs (KLF6, ATF3, JUN, FOS, and FOSB) in various subtypes. The findings showed that FOS expression was considerably elevated in C0 *MAFF*+ TCs relative to those in other subtypes (**Figures 6I, J**). It is still unknown, though, exactly how FOS affects LUAD. Consequently, validating the role of FOS in LUAD cells through *in vitro* functional studies became a principal focus.

### *In vitro* validation through experimental approaches

**Figure 7:**
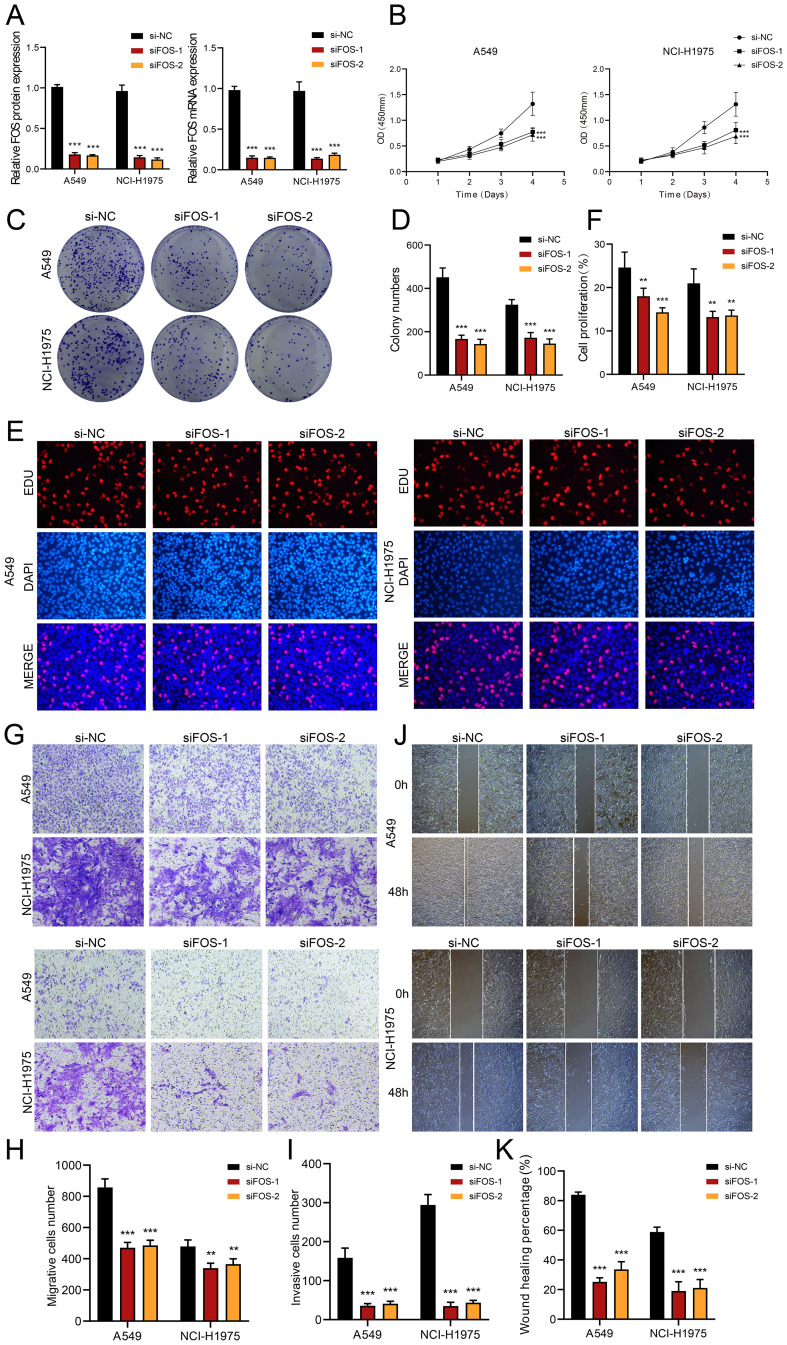
*In vitro* assays demonstrated the consequences of *FOS* knockdown. **(A)** The bar graphs illustrated the levels of genetically encoded proteins (left panel) and gene mRNA (right panel) in the si-NC, si*FOS*-1, and si*FOS*-2 groups within the A549 and NCI-H1975 cells. A notable decline in both mRNA and protein expression followed the reduction of *FOS.* **(B)** The line graph showed that all groups exhibited sustained growth in both cell lines as time progressed. **(C, D)** Colony formation assays indicated that the colony numbers dropped markedly after *FOS* was knocked down, while relevant bar graphs quantitatively displayed colony numbers for all groups in both cell types. **(E, F)** The assay using EDU staining indicated that proliferation was notably inhibited after *FOS* knockdown, as further validated by the bar graphs. **(G-I)** Transwell assays demonstrated that suppressing *FOS* expression led to decreased migration and invasion capacities in both A549 and NCI-H1975 cells. **(J, K)** The cell wound healing assays assessed migration ability in C0 *MAFF*+ TCs after *FOS* knockdown, which led to a statistically significant reduction in wound healing, as illustrated by the respective bar graphs. **P<0.01 and ***P<0.001.

We explored the role of *FOS* in LUAD by performing *in vitro* assays using A549 and NCI-H1975 cell lines. First, we inhibited *FOS* and analyzed the related mRNA and expressions of protein at baseline and post-knockdown. Compared to the control group, both cell lines showed substantially decreased mRNA and protein expression (**Figure 7A**). Furthermore, cell viability was greatly diminished after *FOS* inhibition (**Figure 7B**). We subsequently examined the potential for TCs to proliferate. A significant decrease in cell counts was observed in the colony development assay as a result of the *FOS* suppression (**Figures 7C, D**). In the EDU experiment, we noted a decrease in colony density following the knockdown, data analysis indicated that si*FOS* impeded proliferation (**Figures 7E, F**). To evaluate cell migratory and invasive behaviors, we performed assays of transwell and wound healing. Transwell assays depicted that si*FOS* TCs showed fewer migrated cells and lower cell density compared to the si-NC group, demonstrating that the knockdown of *FOS* impaired their migration and invasion potential (**Figures 7G-I**). The wound healing assays illustrated that si*FOS* inhibited wound healing of TCs in both A549 and NCI-H1975 cell lines (**Figures 7J, K**).

Collectively, the results suggested that reducing *FOS* expression impaired cell activity and significantly decreased the migratory, invasive, and proliferative abilities of TCs. This underlined the important tumor-promoting action of *FOS*, which was essential in the evolution of TCs. Therefore, a main focus of LUAD treatment is *FOS* inhibition since it might increase patient survival and prognosis.

### Developed a prognostic model for LUAD

**Figure 8:**
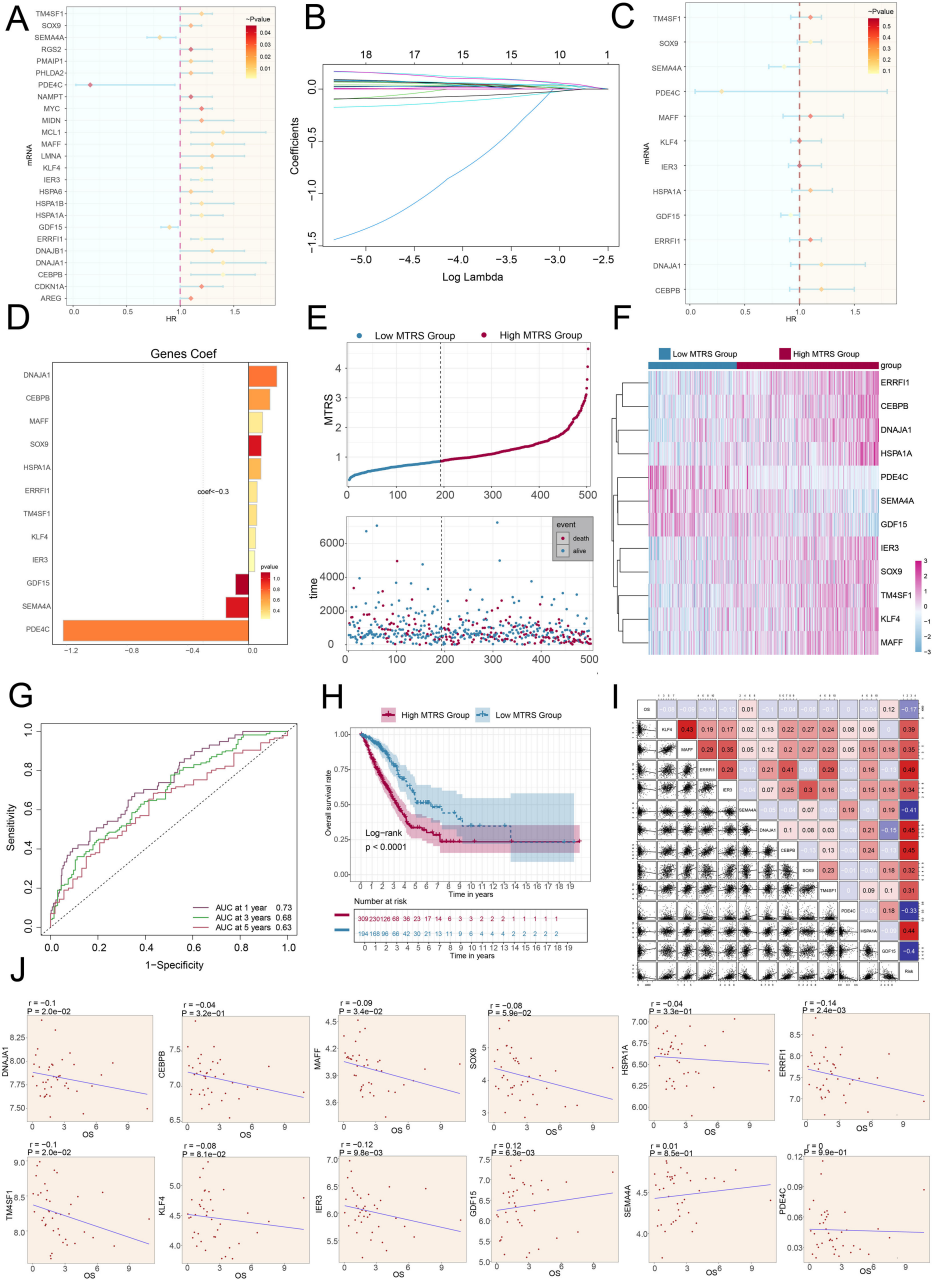
The construction and validation of the model of MTRS. **(A)** The forest plot represented the twenty-five most prognostically significant genes identified by univariate Cox analysis (HR<1 indicated protective factors, whereas HR>1 indicated risk factors.). **(B)** Each plotted line indicated the coefficients attributed to the variables, thereby selecting those with significant prognostic implications. **(C)** The forest plot further presented the top twelve prognostic genes, as determined by multivariate Cox regression. **(D)** The bar graph illustrated the correlation coefficients for these twelve selected genes. **(E)** The curve chart contrasted risk scores between low and high MTRS patient groups, and the scatter plot visualized survival status, marking survival with blue and death with red. **(F)** The heatmap illustrated the expression profiles of twelve risk genes across the two MTRS subgroups. **(G)** ROC curve analysis and its corresponding AUC values assessed the accuracy in predicting survival outcomes in patients. **(H)** The Kaplan-Meier method was applied to evaluate differences in survival between patients with high and low MTRS. **(I)** Both heatmaps and scatter plots visualized correlations among prognostic genes, overall survival, and genes used in constructing the model. **(J)** Scatter plots presented the twelve genes that were associated with overall survival.

We utilized univariate Cox regression analysis to explore prognostic indicators in patients and identified twenty-five genes that exhibited prognosis-related associations. We could observe the HR<1 of *SEMA4A, PDE4C* and *GDF15*, while the HR values of the remaining genes were >1 (**Figure 8A**). To reduce potential multicollinearity across genes, we further refined the list of prognosis-related genes (**Figure 8B**). Following this, we performed multivariate Cox regression analysis on twelve selected genes to determine their respective genetic risk coefficients (**Figures 8C, D**). Additionally, our analysis of the curve chart and scatter plot demonstrated that the low *MAFF*+ tumor risk score (MTRS) group exhibited lower risk scores and better survival outcomes compared to the high MTRS group, which was linked to poorer prognosis (**Figure 8E**). Heatmap further illustrated the distinct prognostic gene expression patterns between these groups, with *SEMA4A, PDE4C*, and *GDF15* more strongly expressed in the low STRS group, suggesting their potential positive impact on prognosis (**Figure 8F**). Moreover, the ROC curves and corresponding AUC values at 1-year, 3-years, and 5-years were 0.73, 0.68 and 0.63 respectively, highlighting the strong predictive power of the model (**Figure 8G**). Kaplan-Meier survival analysis further corroborated the worse prognosis observed in the high MTRS group (**Figure 8H**). In summary, our analysis revealed a negative association between the risk score and survival, supporting the observation that higher risk scores were linked to shorter survival periods (**Figure 8I**).

Further examination of the expression levels of twelve prognostic genes in the high MTRS group and low MTRS group, the scatter plots showed that *GDF15* and *SEMA4A* were beneficial for patient prognosis, while *DNAJA1, CEBPB, MAFF, SOX9, HSPA1A, ERRFI1, TM4SF1, KLF4*, and *IER3*were associated with poor patient prognosis (**Figure 8J**, **Supplementary Figures 2A, B**).

### Analyses included the investigation of immunoinfiltration, enrichment assessment, and examination of drug sensitivity

**Figure 9:**
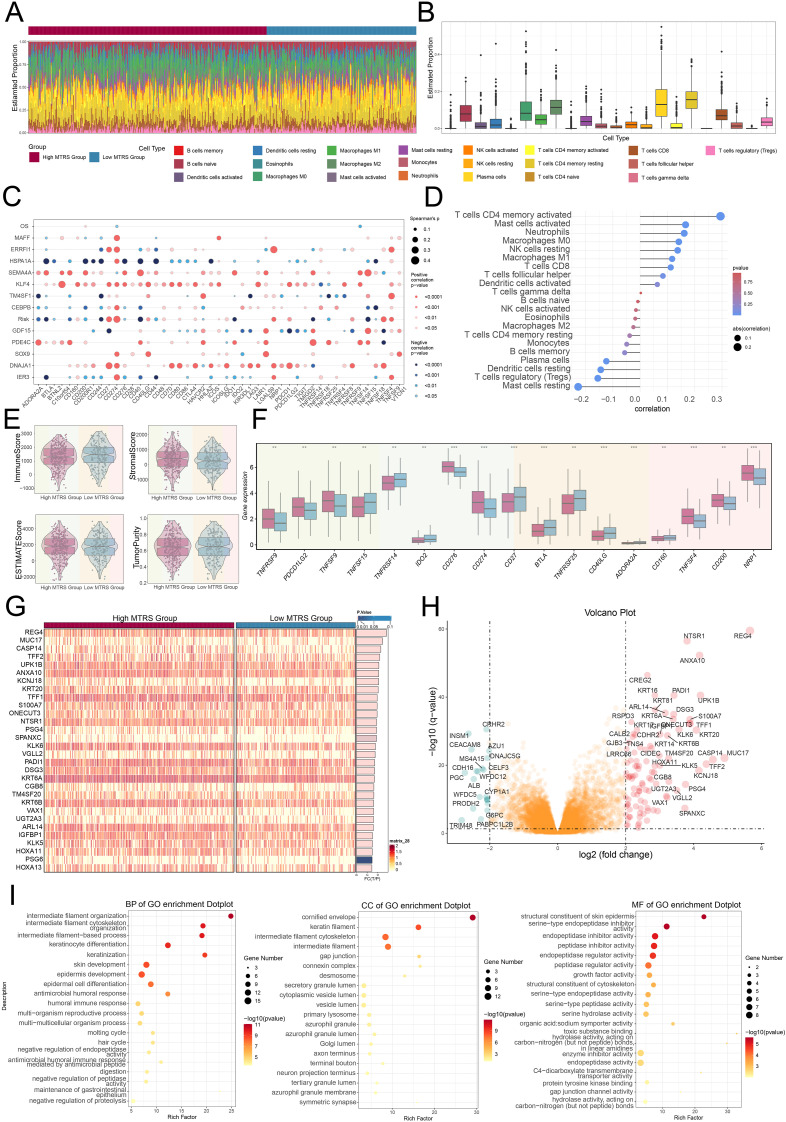
Immunoinfiltration differences and enrichment analysis. **(A, B)** Stacked bar graph, along with the box plot, demonstrated the calculated distributed twenty-two immune cell types across various risk score groups. **(C)** Bubble plot illustrated the strength of correlation that existed between risk genes and immune checkpoints. **(D)** Lollipop plot reflected the correlations between multiple immune-related pathways and risk scores, where bubble diameter indicated the magnitude of association and color signified the statistical significance (p-value). **(E)** The violin plots showed comparisons of immune score, stromal score, estimate score, and tumor purity between the high and low MTRS groups. **(F)** The box plot revealed significant differences in immune checkpoint expression when comparing high MTRS group to low MTRS group. **(G)** The heatmap showed that gene expression patterns varied distinctly between the two MTRS groups. **(H)** The volcano plot provided a visual summary of expression changes among DEGs. **(I)** The dot plots presented the GO enrichment results for BP, CC, and MF categories, respectively. **P< 0.01 and ***P< 0.001..

The study compared gene expression levels and underlying biological processes across groups with high and low values, we made use of various visualization and enrichment analysis techniques. We first illustrated the estimated proportions of cell populations in both groups using a stacked bar graph (**Figures 9A, B**). Additionally, we utilized a bubble plot to illustrate the correlations among immune checkpoints, prognostic genes, overall survival, as well as the risk, revealing that KLF4 generally exhibited a positive correlation with immune checkpoint-associated genes, while HSPA1A showed a negative relationship (**Figure 9C**). Upon additional examination, we observed that higher risk scores were correlated with increased macrophage presence (**Figure 9D**). Moreover, the stromal and estimate appeared higher in the high MTRS group, while the immune score was lower compared to the low MTRS group (**Figure 9E**). Through box plot analysis, we discovered that the majority of immune checkpoint genes had considerably increase expression in the high MTRS group as opposed to the low group (**Figure 9F**).

To shed light on the differences between the high and low scoring cohorts, we examined genes that were differentially expressed (**Figure 9G**). The volcano plot further depicted the trends of gene upregulation and downregulation among these DEGs (**Figure 9H**). Following this step, enrichment analysis was undertaken to explore the biological processes associated with the identified genes. We initially performed GO enrichment analysis, which identified significantly enriched terms in biological processes (BP), cellular component (CC), and molecular functions (MF) (**Figure 9I**). In terms of GO-BP, the genes were prominently enriched in pathways involved the organization of intermediate filaments, the structuring of the intermediate filament cytoskeleton, processes based on intermediate filaments, and the differentiation of keratinocytes. The cornified envelope, keratin filaments, intermediate filament cytoskeleton, and intermediate filaments were identified as the main sites of enrichment in terms of GO-CC. GO-MF analysis revealed enrichment included structural constituent of the skin epidermis as well as serine-type endopeptidase inhibitor, endopeptidase inhibitor, and peptidase inhibitor activities.

**Figure 10:**
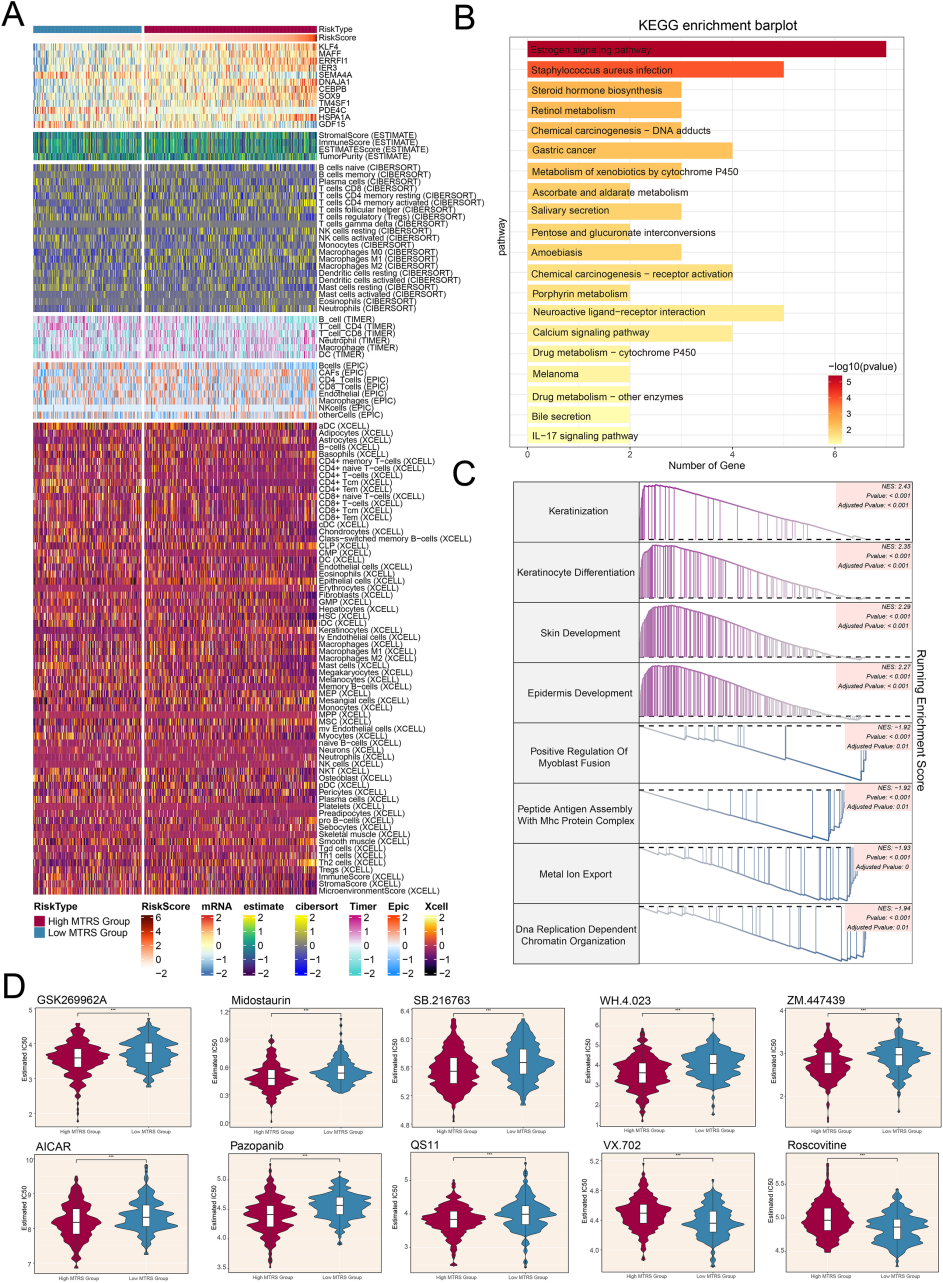
Differences in immune cell infiltration, results of enrichment analysis, and findings from drug sensitivity assessments across various risk groups. **(A)** Heatmap illustrated how risk scores of different immune cell types varied between the high and low MTRS groups. **(B)** The KEGG enrichment bar plot reported the twenty most significantly enriched pathways. **(C)** The GSEA enrichment analysis thoroughly summarized the gene sets that distinguished the high MTRS group from the low MTRS group. **(D)** Violin plots highlighted marked disparities in the IC50 values for different chemotherapeutic agents between patients belonging to the high and low MTRS categories. ***P<0.001..

Afterwards, the heatmap offered a clear visual comparison that further supported our earlier findings (**Figure 10A**). Additionally, we performed KEGG pathway analysis, which highlighted enrichment in pathways such as the estrogen signaling pathway, staphylococcus aureus infection, steroid hormone biosynthesis, and retinol metabolism (**Figure 10B**). To better elucidate the functionality and enrichment of pathway associated with LUAD, further investigations were carried out, we also conducted GSEA analysis (**Figure 10C**). These results demonstrated positive enrichment trends in keratinization, differentiation of keratinocytes, development of the skin, and the epidermis underwent development. Conversely, negative enrichment trends were observed for positive regulation of myoblast fusion, peptide antigen assembly with MHC protein complex, metal ion export, and DNA replication-dependent chromatin organization.

In conclusion, the results of our drug sensitivity analysis indicated that the high MTRS group showed heightened sensitivity to chemotherapeutic agents (**Figure 10D**), including GSK269962A, Midostaurin, SB.216763, WH.4.023, ZM.447439, AICAR, Pazopanib, and QS11. Furthermore, it was determined that the low MTRS group exhibited decreased IC50 values for VX.702 and Roscovitine compared to the high MTRS group. This finding suggested that VX.702 and Roscovitine may have more favorable therapeutic effects for low MTRS group patients.

## Discussion

Lung cancer is the most prevalent malignant tumor in both China and the world, with the highest incidence and mortality rates. Among its various pathological types, LUAD is the most common, accounting for approximately 40% of lung cancer cases (3). The anti-tumor efficacy in LUAD patients is often limited by chemotherapy resistance and evasion of apoptosis, leading to tumor recurrence and poor prognosis (56, 57). As a result, the survival duration for LUAD patients has been notably poor. Therefore, it became urgent to develop personalized treatments based on the progression of LUAD, necessitating a comprehensive investigation of the tumor’s heterogeneity to improve patient outcomes and identify potential therapeutic targets. Through scRNA-seq analysis, the characteristics related to both cells and molecules in LUAD tissue were studied, revealing fourteen distinct cell types. Temporal phase, sample origin, and pathway enrichment analyses were conducted for the different cell subtypes. The IAC group primarily contained EPCs and macrophages, suggesting that these cell types may play a key role in the development of malignant tumors. Tumors were composed of various cell types and components that competed with the normal microenvironment. Consequently, the interactions among these cell types might have facilitated the tumor’s aggressiveness and resistance to treatment (58).

Enrichment analysis revealed that EPCs exhibited positive enrichment in pathways associated with chemical homeostasis within a tissue and surfactant homeostasis. Pulmonary surfactant, a complex of lipids and proteins, served a key function in decreasing the interfacial tension at the alveolar air-liquid interface, and its production is regulated during development (59). Genes related to pulmonary surfactant have been regarded as excellent candidate genes for lung cancer risk due to their high expression levels in the lungs and their role in maintaining alveolar integrity (60). Studies demonstrated that the disruption of pulmonary surfactant homeostasis may be linked to the onset of lung cancer, with abnormalities in the genes and cellular processes related to surfactant maturation and function identified as risk factors for lung cancer (61–64). During the process of self-evolution, cancer cells give rise to a class of tumor stem cells characterized by self-renewal, metastatic spread, and treatment resistance, functioning as a major driver in cancer initiation and subsequent growth (65).

Accordingly, we conducted a study focused on TCs within LUAD. Marker gene profiles were used to assign tumor tissues to one of four subtypes in the initial step. Notably, it was observed that TCs with increased *MAFF* expression were mainly derived from the IAC group. *CXCL8* was initially identified as an effective chemoattractant and activator of polymorphonuclear leukocytes. It also influenced cancer cell proliferation, migration, tumor angiogenesis, and metastasis, being expressed in various cancer cell types (66). *CXCL2*, a small secretory member of the CXC chemokine family (67), played a critical role in maintaining macrophage infiltration induced by cancer (68). *RRAD* was associated with multiple cancer types and significantly contributed to regulating aerobic glycolysis and cellular invasion and metastasis (69). *ATF3* having a significant impact on regulating metabolism, immunity, and tumorigenesis (70). *AREG* promoted cellular proliferation in various tumors and inhibited apoptosis while a vital contribution was made in the extracellular matrix environment or bloodstream (71). The synergy among these genes effectively demonstrated the C0 *MAFF*+ tumor cell subtype’s role in promoting tumorigenesis. Furthermore, the C0 *MAFF+* tumor cell subtype displayed elevated levels of nCount RNA, nFeature RNA, and stemness expression, indicating a higher degree of malignancy and differentiation potential for these cells. All things considered, there was a complex interaction between C0 *MAFF*+ TCs and the development of LUAD.

We carried out a number of cell subtype enrichment analyses in order to elucidate the association between cancer and C0 *MAFF*+ tumor cell subtype. The biological processes associated with the response to lipid and fat cell differentiation were the main ones in which C0 *MAFF*+ TCs participated. The metabolic activity of cancer cells often exhibited specific alterations, with this metabolic reprogramming supporting an increase in metabolic intermediates necessary for the synthesis of proteins, nucleic acids, and lipids, which are prerequisites for the rapid proliferation of cancer cells. The increased rate of lipid synthesis in tumor tissues has long been recognized as a significant aspect of the metabolic rewiring of transformed cells (72). Abnormal lipid synthesis and extracellular lipid uptake serve as beneficial modifications to meet the demands of uncontrolled cancer cell proliferation (73).

To rephrase, LUAD TCs tended to grow faster when supported by lipids, which was also linked to a higher degree of malignancy. The metabolic interactions between TCs and adipocytes induced the mesenchymal transformation of adipocytes and contributed to the reconfiguration of the stroma into a microenvironment more conducive to tumors. Regions of tumor-adipocyte interactions exhibited high densities of angiogenesis, indicating that TCs were in a more active state due to the nutrient-rich environment in these areas (74). To summarize, processes like lipid metabolism and adipocyte differentiation were key contributors to tumor formation and advancement, potentially promoting the rapid growth and spread of TCs and heightening tumor malignancy. In short, intervening in factors such as lipid metabolism or fat cell differentiation might have slowed the progression of LUAD.

The pseudotime analysis revealed that the C0 subtype was located at the terminal end of lineage 1, and the C0-associated gene *MAFF* was highly expressed primarily in the later stages, indicating a more advanced developmental state and a mature phase of differentiation. The high level of cellular stemness suggested a low differentiation status for C0 subtype, which correlated with higher malignancy and stronger resistance to chemotherapy and radiotherapy. This, in turn, leads to poor prognosis and a higher likelihood of recurrence in patients.

To further investigate the interactions between C0 *MAFF+* TCs and other cells, CellChat identified that C0 *MAFF+* TCs act on macrophages via the MIF-(CD74+CD44) signaling pathway. MIF, known as Macrophage Migration Inhibitory Factor, was a cytokine expressed in various cell types, including HCs, EPCs, ECs, MCs, and NCs. Altered expression of MIF was linked to various diseases, extending from inflammatory illnesses to organ abnormalities and different forms of malignancy (75). Enhanced expression of MIF in tumor tissues suggested a potential oncogenic role of macrophages (76). Studies have shown that the MIF-CD74 signaling pathway promotes tumor cell proliferation (77). It served as a crucial cytokine in the context of both tumorigenesis and inflammation, MIF triggers MAPK and PI3K signaling pathways by binding to the CD74 receptor (78). Activation of MAPK and PI3K pathways by MIF regulated fundamental cellular functions related to proliferation, differentiation, apoptosis, cell survival, and cancer development (79). In normal lung tissue, MIF mRNA and protein were expressed in bronchial epithelium, alveolar epithelium, vascular smooth muscle, and alveolar macrophages. In LUAD tumor tissues, levels of MIF mRNA and protein were significantly higher than those in normal alveolar EPCs, with elevated MIF mRNA levels in both TCs and premalignant states in LUAD (80). These findings indicated that high expression of MIF promotes tumor growth and metastasis in LUAD and created a TME conducive to tumor development. C0 *MAFF+* TCs might promote the transformation of normal macrophages into TAMs by acting on the CD74-CD44 receptor of macrophages via the MIF ligand, thus, it blocked the natural immune response that targeted TCs. TAMs directly influenced tumor cell communication by transferring substances such as some non-coding RNAs through exosomes, which affected TCs (81). Additionally, TAMs induced immune checkpoint inhibition of T cells by upregulating PD-L1 expression and recruited Tregs via CCL22 to further suppress anti-tumor immune responses (82). TAMs primarily exhibited M2-like tumor-promoting effects within the TME and regulated various malignant processes, including angiogenesis, immunosuppression, and tumor metastasis (83). Therefore, targeting M2-like TAMs to deplete them in the TME or reversing M2-like TAMs to an M1-like phenotype directly enhanced their cytotoxicity and indirectly stimulated cytotoxic T cells to eliminate TCs, representing a potential strategy for anti-tumor immunotherapy (84, 85). Thus, inhibiting MIF within TCs emerged as a promising avenue for therapeutic intervention.

For a deeper understanding of the oncogenic mechanisms of the C0 *MAFF*+ tumor cell subtype, we analyzed the TFs within this subtype and identified the top five active TFs: KLF6, ATF3, JUN, FOS, and FOSB.

Members of the KLF protein family play vital roles in regulating key biological activities such as cell growth, specialization, metabolic regulation, programmed cell death, and inflammations. Aberrant KLF function can disturb cellular equilibrium and has been implicated in the pathogenesis of various diseases. KLF6 was implicated in cancer, inflammatory diseases, and cardiovascular disorders (86). As a TF induced by stress, ATF3 was vital for modulating metabolism, immune responses, and tumorigenesis. ATF3 expression was promoted by several extracellular stimuli, among them endoplasmic reticulum stress, cytokines, and chemokines. Additionally, ATF3 served as a major regulator of metabolic homeostasis (70). JUN belonged to the most widely analyzed elements of the AP-1 complex and was engaged in numerous activities, including cell division, programmed cell death, survival, cancer development, and tissue formation. Early studies identified JUN as a basic leucine zipper TF that functioned as a homo- or heterodimer to bind DNA and regulated gene transcription. Subsequent research demonstrated that extracellular signals can induce post-translational modifications of JUN, leading to altered transcriptional activity and target gene expression (87). Members of the FOS protein family could be divided into two groups: transforming (c-Fos and FosB) and non-transforming (Fra-1 and Fra-2) proteins (88). FOS encoded leucine zipper proteins that dimerize with proteins from the JUN family to form the TF complex AP-1, which played a key role in tumor cell growth, differentiation, survival, and DNA damage response (16, 17). Notably, overexpression of FOS could promote drug resistance and enhance EMT (89, 90). FOSB, also known as FBJ murine osteosarcoma viral oncogene homolog B, was a member of the FOS TF family (91). FOSB was an oncogene present in various tumors that promoted angiogenesis and regulated genes associated with drug sensitivity and invasive activity (92, 93). In summary, these findings provided innovative perspectives for future immunological interventions in LUAD.

To improve patient survival rates, enhance their quality of life, and prolong longevity, our study constructed a prognostic risk prediction model for LUAD by employing the top twelve marker genes of C0 *MAFF*+ TCs. We observed that the high MTRS group exhibited higher risk scores, indicating increased mortality rates and poorer prognoses. The results suggested that *FOS* contributed to the progression of LUAD and was generally correlated with adverse outcomes in cancer patients.

*In vitro* assays demonstrated that knocking down *FOS* greatly diminished the abilities of TCs to proliferate, migrate, and invade. Overexpression of *FOS* had been shown to promote drug-resistant phenotypes (89). Therefore, considering *FOS* targeting as a viable strategy to hinder these carcinogenic processes and improve treatment outcomes in LUAD patients appeared promising. Future studies could explore the combination of *FOS*-targeted therapies with existing treatments to enhance the efficacy of LUAD immunotherapy. Moreover, analysis of immune cell infiltration demonstrated that the high MTRS group exhibited significantly increased stromal scores, estimate scores, and levels of macrophage infiltration in comparison to the low MTRS group. Further examination of the associations in the relationship of MTRS to immune infiltration showed that MTRS was significantly and positively correlated with activated CD4 memory T cells, activated mast cells, and M1 macrophages, whereas it was negatively associated with M2 macrophages and resting mast cells. In most cases, macrophages were divided into two principal subtypes: the classically activated M1 and the alternatively activated M2 (94). M1 macrophages played a role in mediating resistance against tumors. In contrast, various forms of M2 macrophages were present in established tumors and promoted progression, tissue repair, remodeling, and exhibited immunoregulatory functions (95). The higher levels of macrophage infiltration in the high MTRS group favored the targeting of macrophages in LUAD patients, facilitating the occurrence of normal anti-tumor effects. Targeting M2 macrophages might be a feasible therapeutic approach, and enhancing M1 macrophages could further improve patient prognoses.

In our continued efforts to elucidate the function of macrophage phenotype control in anti-cancer therapy, with emphasis on promoting the transition from M2 to M1 macrophages, analysis indicated that STING agonists promoted the local production of anti-angiogenic factors and normalized tumor-associated vasculature (96), suggesting that reprogramming macrophages into anti-tumor states was promising. Additionally, previous research showed that tetrahedral DNA nanostructures actively entered macrophages to enhance M1 polarization (97), while mitochondrial DNA induced macrophage recruitment and M2 polarization through the TLR9 pathway, inhibiting this pathway reversed mitochondrial DNA-mediated M2 macrophage polarization (98). These approaches effectively suppressed M2 polarization and promoted M1 activation, highlighting the potential benefits of combining immunomodulatory therapies in LUAD. In our study, the group with elevated MTRS showed notably worse prognoses in comparison to the low MTRS group, therefore, therapeutic strategies targeting the polarization of TAMs from the M2 to the M1 might have offered significant benefits Furthermore, targeting *FOS* might enhance the immunotherapeutic response in patients with either high or low MTRS. Therefore, *FOS* could serve as a novel target for improving the reprogramming of macrophages. Later studies might have assessed the combination of *FOS*-targeted therapies with existing treatments to improve macrophage-based cancer immunotherapy outcomes.

Furthermore, we analyzed the samples for their sensitivity to drugs across different risk score groups to uncover potential differences, which also contributed to the development of personalized treatment strategies. Our study revealed that GSK269962A, Midostaurin, SB.216763, WH.4.023, ZM.447439, AICAR, Pazopanib, and QS11 demonstrated stronger efficacy in patients within the high MTRS group.

AICAR was a pharmacological precursor in purine nucleotide biosynthesis with antitumor properties (99). AICAR has long been one of the most commonly used pharmacological regulators of AMPK activity, and most early studies on the role of AMPK in metabolic regulation and cancer pathogenesis were entirely based on the use of AICAR as an AMPK activator (100). It was found that patients belonging to the high MTRS category exhibited higher sensitivity to AICAR. Studies have indicated that GSK269962A was a selective ROCK1 inhibitor (101), which can inhibit tumor growth (102).

Midostaurin was a multitarget kinase inhibitor initially developed as a protein kinase C inhibitor for the treatment of solid tumors (103). SB.216763, a drug that functioned as a Wnt signaling pathway suppressor, was shown to suppress the proliferation, migration, and invasion of TCs (104). WH.4.023 was a chemotherapy drug that significantly targeted the proto-oncogene tyrosine-protein kinase SRC and the tyrosine-protein kinase ABL1 (105, 106). ZM.447439 was an Aurora selective ATP-competitive inhibitor that could disrupt spindle integrity checkpoints and chromosome segregation, making it useful for more selective cancer treatment (107). Pazopanib was an orally administered small-molecule multitarget kinase inhibitor that inhibited angiogenesis induced by vascular endothelial growth factor and basic fibroblast growth factor, and could be used as a treatment for tumors (108). QS11 influenced protein transport by inhibiting the GTPase-activating protein ARFGAP1, thereby co-activating the Wnt/β-catenin signaling pathway. When overexpressed in breast cancer cells, it inhibited cell migration, indicating that QS11 might have potential applications in tumor regulation (109). Our findings highlighted the differences in drug sensitivity among LUAD patients, such findings emphasized the importance of conducting additional studies to better understand how they functioned, enhanced efficacy, and developed personalized treatment strategies to improve patient prognosis.

In our research, although we performed a comprehensive evaluation of LUAD and meticulously selected our study population, we recognized several shortcomings. Firstly, given the restricted sample size, it is possible that this study encountered errors in correlating the examined samples with the target genes, thereby possibly influencing the accuracy of the analysis. Furthermore, the limited number of samples might have resulted in imprecisions that could challenge the reliability of the conclusions derived from the research. Secondly, LUAD comprised multiple subtypes. Yet, considering the high prevalence of LUAD in lung cancer, our investigation’s specificity was possibly limited, yielding more generalized outcomes since different LUAD subtypes could possess unique features and respond differently to treatments. Finally, our experimental assessment of the interplay between TCs and macrophages was not as detailed as desired. TAMs and other TME cellular components co-evolved with TCs, so fostering tumor progression and resistance and contributing to tumor heterogeneity. Future studies should thus concentrate more on these interactions and their effect on tumor growth. We intended to start research on *FOS* and investigate cell-targeted therapy approaches against LUAD in next projects. Clarifying the *FOS* mechanisms in LUAD would help us to create fresh therapeutic approaches to raise patient prognosis. We also sought to investigate how different TME-targeting techniques might be combined into a coherent approach to improve efficacy and lower side effects, so offering more treatment choices for LUAD patients.

## Conclusion

Driving lipid metabolic reprogramming and immunosuppression through MIF-(CD74+CD44)-mediated macrophage polarization, our work finds *FOS* as a master regulator of the malignant C0 *MAFF*+ tumor cell subtype in LUAD. The MTRS prognostic model stratifies patients rather well, high-risk cases show increased chemosensitivity to AICAR and Midostaurin. *In vitro*, functionally *FOS* knockdown suppressed proliferation, migration, and invasion, so highlighting its therapeutic potential. These results suggest *FOS* inhibition as a dual approach to target immune evasion and tumor-intrinsic malignancy so improving LUAD treatment. To maximize clinical benefit, future research should look at *FOS*-directed treatments in concert with immune checkpoint inhibitors.

## Data Availability

The original contributions presented in the study are included in the article/**Supplementary Material**. Further inquiries can be directed to the corresponding authors.

## Supplementary material

Supplementary Figure 1 InferCNV analysis. **(A)** The prediction of CNVs was performed using scRNA-seq data sourced from ECs, and orange was used to indicate amplification, whereas blue signified deletion.

Supplementary Figure 2 The analysis and expression levels of prognostic genes. **(A, B)** The Kaplan-Meier survival analysis illustrated which prognostic genes were associated with C0 *MAFF*+ TCs, while the gene expression profiles were further examined in both high and low MTRS groups.
