# Supplementary figures and images for: Targeted and personalized immunotherapy in lung adenocarcinoma: single-cell RNA sequencing of MAFF+ tumor cells and the therapeutic potential of FOS
Source: Front Immunol. 2025 Aug 27;16:1649147. doi: 10.3389/fimmu.2025.1649147 (PMC12420628; doi:10.3389/fimmu.2025.1649147)

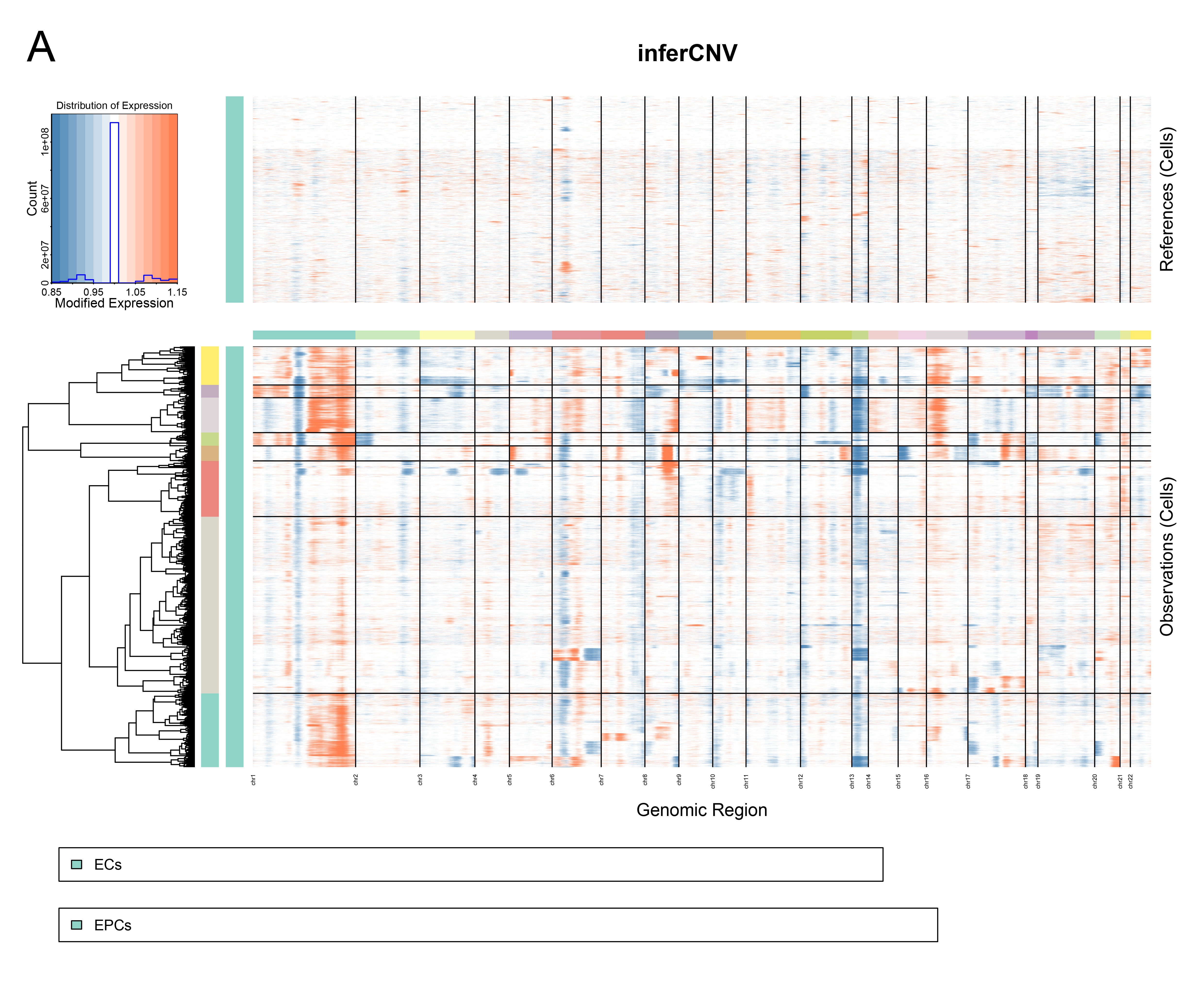

Supplement: Supplementary Figure 1 — InferCNV analysis. (A) The prediction of CNVs was performed using scRNA-seq data sourced from ECs, and orange was used to indicate amplification, whereas blue signified deletion. [file Image1.jpeg]

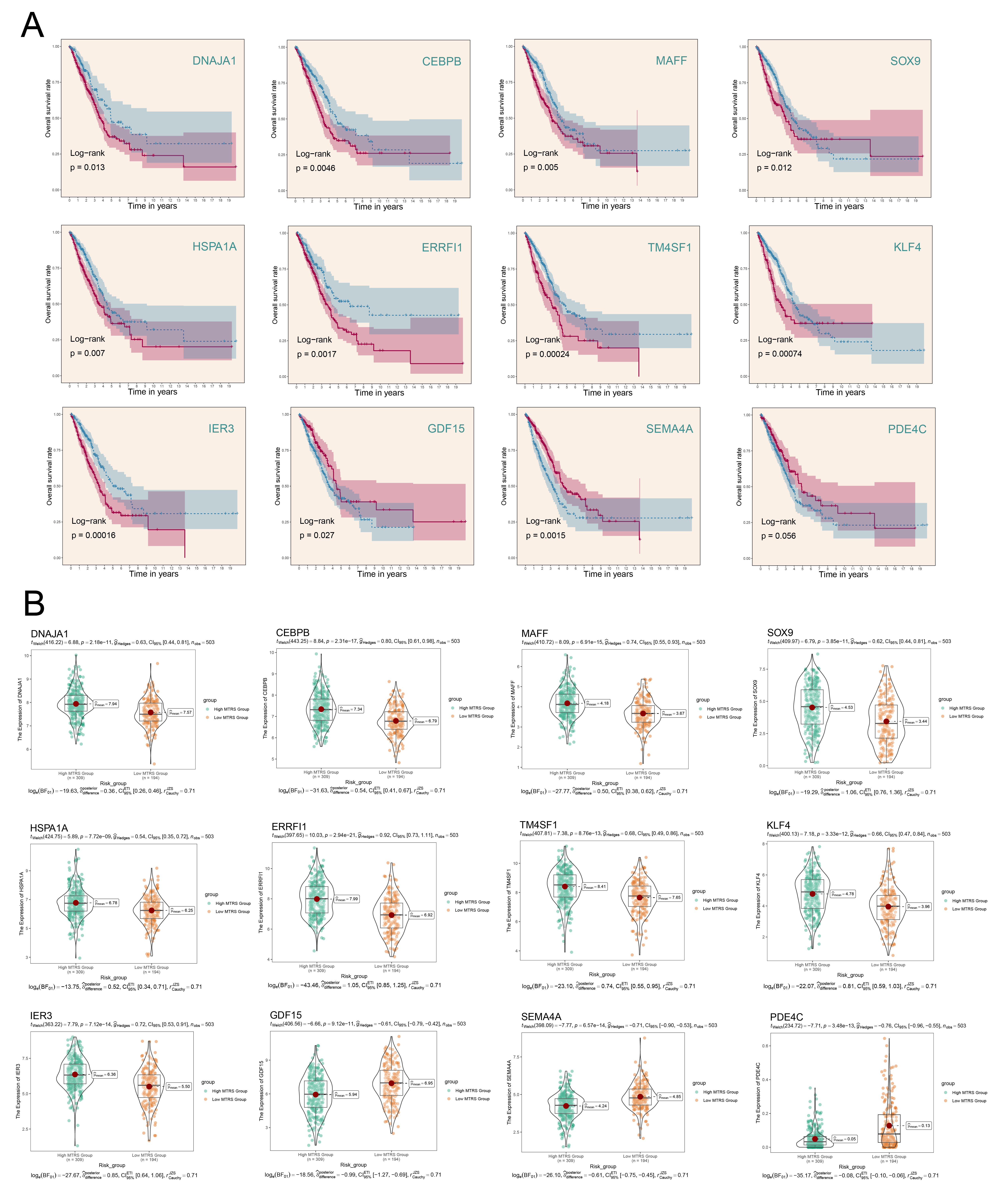

Supplement: Supplementary Figure 2 — The analysis and expression levels of prognostic genes. (A, B) The Kaplan-Meier survival analysis illustrated which prognostic genes were associated with C0 MAFF+ TCs, while the gene expression profiles were further examined in both high and low MTRS groups. [file Image2.jpeg]
